# Dynamical climatic model for time to flowering in Vigna radiata

**DOI:** 10.1186/s12870-020-02408-1

**Published:** 2020-10-14

**Authors:** Konstantin Kozlov, Alena Sokolkova, Cheng-Ruei Lee, Chau-Ti Ting, Roland Schafleitner, Eric Bishop-von Wettberg, Sergey Nuzhdin, Maria Samsonova

**Affiliations:** 1 Peter the Great St. Petersburg Polytechnic University, 29 Polytechnicheskaya, St.Petersburg, 195251 Russia; 2 National Taiwan University, Taipei, Taiwan; 3 World Vegetable Center, Tainan, Taiwan; 4 Department of Plant and Soil Science, University of Vermont, 63 Carrigan Drive, Burlington, VT, 05405 USA; 5 Program Molecular and Computation Biology, University of California, University Park, Los-Angeles, CA, 24105 USA

**Keywords:** Vigna, Mungbean, Model, Climatic factors, GWAS

## Abstract

**Background:**

Phenology data collected recently for about 300 accessions of *Vigna radiata* (mungbean) is an invaluable resource for investigation of impacts of climatic factors on plant development.

**Results:**

We developed a new mathematical model that describes the dynamic control of time to flowering by daily values of maximal and minimal temperature, precipitation, day length and solar radiation. We obtained model parameters by adaptation to the available experimental data. The models were validated by cross-validation and used to demonstrate that the phenology of adaptive traits, like flowering time, is strongly predicted not only by local environmental factors but also by plant geographic origin and genotype.

**Conclusions:**

Of local environmental factors maximal temperature appeared to be the most critical factor determining how faithfully the model describes the data. The models were applied to forecast time to flowering of accessions grown in Taiwan in future years 2020-2030.

## Background

Among the cultivated species in the legume family, mungbean (*Vigna radiata* (L.) Wilczek), also known as green gram) has become one of the important crops across Asia and beyond, showing a steady increase in global production (FAO 2018). This short duration legume crop fits easily as a rotation crop into cereal based production systems, adding nitrogen to the soil for the following crop, and providing additional income for farmers. Mungbean is a valuable source of protein and contains high amounts of the essential micronutrients folate and iron. Beyond the agronomic value of mungbean, certain features make it a well-suited model organism among legume plants due to its relatively small genome size, short life-cycle, self-pollination, and close genetic relationship to other important legume crops. Mungbean is often produced in marginal areas or during hot seasons, where abiotic stress limits its productivity. Mungbean yellow mosaic disease (Begomovirus strains), which is transmitted by whitefly (*Bemisia tabaci*) has significant impacts on yields as well harvests [1].

Biodiverse collections of mungbean and related species are available in various genebanks, e.g. at the World Vegetable Center (Taiwan), in the National Bureau of Plant Genetic Resources (India), in the Institute of Crop Germplasm Resources (China), the Plant Genetic Resources Conservation Unit (USA), the genebank of the Commonwealth Scientific and Industrial Research Organization (Australia), and Plant Genetic Resources Program (Pakistan). The collection at the N.I. Vavilov All-Russian Institute of Plant Genetic Resources (Russia) contains 1,478 accessions of *V. radiata* and 230 of *V. mungo* [2, 3]. Various core collections have been established to improve the access to mungbean biodiversity in breeding [4].

It has been shown that high yielding mungbean varieties should possess larger leaf area, higher total dry mass production ability, superior crop growth rate at all growth stages, and high relative growth rate and net assimilation rate at vegetative stage which would result in superior yield components [5]. Analysis of international mungbean trials suggested strong genotype-by-environment (G x E) interactions [6] some of which were related to physiological traits including time to flowering and maturity. Time to flowering in mungbean is subject to both genetic [7], and environmental control [8]. Inconsistency of seed yield often experienced in mungbean, is due to its differential response of genotypes to various growing season or conditions. In general, productivity of a plant is influenced by management, and in addition by several factors such as climate, soil type, photoperiodic response and micro-environments. Thus, the significance of genotype x environment interaction for obvious reason deserves high priority in any crop improvement program. Promising genotypes need to be evaluated in multi-environmental tests over several years for identification of the stable and widely adapted genotypes [9].

Molecular markers are routinely utilized worldwide in all major crops as a component of breeding. The pace of development of molecular markers, establishment of marker-trait associations for important agronomic traits has been accelerated breeding in other pulses [10], but so far, progress in marker-assisted selection as a part of mungbean breeding programmes has been very limited [11].

In the past, there have been several efforts to develop molecular markers and linkage maps associated with agronomic traits for the genetic improvement of mungbean and, ultimately, breeding for cultivar development to increase the average yields of mungbean. The recent release of a reference genome of the cultivated mungbean (*V. radiata var. radiata* VC1973A) and an additional de novo sequencing of a wild relative mungbean (*V. radiata var. sublobata*) has provided a framework for mungbean genetic and genome research that can be expanded for genome-wide association and functional studies to identify genes related to specific agronomic traits [12, 13]. Moreover, the diverse gene pool of wild mungbean comprises valuable genetic resources of beneficial genes that may be helpful in widening the genetic diversity of cultivated mungbean [3, 12].

To effectively harness molecular markers, crop phenology models that integrate genotypic variation can be critical tools [14]. A number of simulation models have been developed for other species of cultivated *Vigna*, as well as mungbean. Bambara groundnut (*Vigna subterranea*), an important oil seed crop that is phenotypically similar to groundnut has been examined with the AquaCrop model [15]. While the results of these simulations are preliminary, they confirm the view that bambara groundnut is a potential future crop suitable for cultivation in marginal agricultural production areas. Future research should focus on crop improvement to improve current yield of bambara groundnut [15]. The APSIM model could be utilised to characterise growing environments which could in turn be used to minimise other contributors to G x E interactions, including managing phenology to match target production environments. This approach has been used in sorghum [16]. However, the APSIM model needs further improvement and validation for its future use to assist breeding programs [17].

However the models developed in the pre-genomic era considered genotype influence at best as a set of given “genetic coefficients” that do not correspond to actual genes [18]. Mathematical models and tools that combine genetic and climate data to predict agronomic traits will greatly benefit breeders by simulating the performance of any given well-characterized genotype in any given well-characterized environment [19, 20].

Natural selection shapes genetic variation of a population and thereby determines local adaptation [21]. The signatures of adaptations in genome can be revealed by association between environmental conditions at a site of an accession’s origin and SNPs [22]. GWAS is a good method to identify associations between genomic regions and traits, however its design usually requires controlled planting of replicated accessions. This can quickly become logistically challenging and expensive, especially in multi-environmental trails. Crop models may complement GWAS approaches by accounting for the influence of environment at accession sampling sites.

In this work we built a new dynamical model for time to flowering in 296 accessions of mini core collection of *Vigna radiata* phenotyped during four field trials in different years and two locations. We further used the model to investigate the effects of adaptation of genotypes to environmental factors. Finally, we forecast the time to flowering for the years 2020-2030 using generated daily weather.

## Results

ANOVA test applied to the whole dataset showed that the differences in mean time to flowering between countries of origin are statistically insignificant with criterion value *F*=1.383 and *p*=0.125. Consequently, time to flowering is not explained by a simple linear dependence on the country of origin and climatic control functions are to be found.

To find parameters of models (15) and (17) we performed for each model 10 optimization runs of Differential Evolution Entirely Parallel method for each value of *λ*=1,10,50,100,150,200,300,500,1000,1500,2000. Different seeds for random number generator were used for each run.

### A model with the country of origin information

**Fig. 1:**
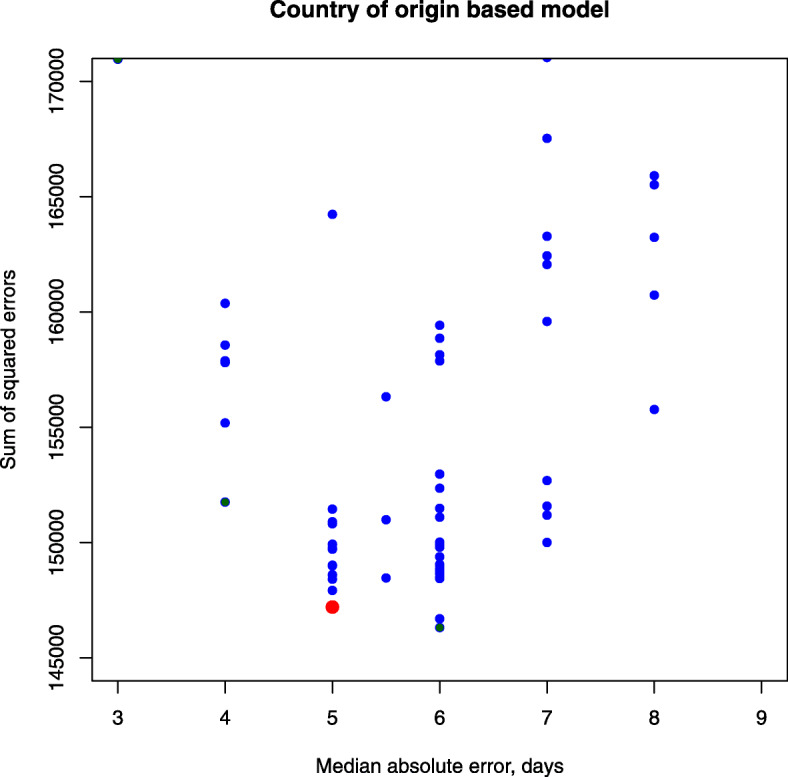
Models with country of origin information. Each model is represented with a dot and the best one is marked as large red dot

To select the best model for further investigation we compared model and data and plotted the sum of squared differences in time to flowering against median absolute difference in this trait (see Fig. 1). The best model should minimize both criteria and hence corresponds on the graph to the closest to the origin dot at the bottom left corner.

The selected model is defined by (1)–(6). In this model the average error (root mean square) in time to flowering prediction is 11.6 days (*λ*=50), the resource amount Y needed to flower is 40.27 and the coefficient threshold *B*_*min*_=0.63.

1 $$ {}{\begin{aligned} \Delta y(i,t) &=0.83\cdot F_{0} + 4.45\cdot F_{1} + 0.13\cdot F_{2} + 9.57\cdot F_{3}\\ &+ 5.75\cdot F_{4} + \sum\limits_{n=0}^{4}\Theta_{n} F_{n}\\ F_{0} &= \frac{rain}{dl - 12.995}\\ F_{1} &= \left((dl - 12.995) - \frac{1}{dl - 12.995} \right)\cdot \frac{1}{tmin - 0.109768} \\  F_{2} &= \left(rain + \frac{1}{tmax - 29.9983} \right) \\ F_{3} &= \frac{1}{rain - 20.582} \\ F_{4} &= \left(\frac{1}{srad - 2.60235} + \frac{1}{tmin - 0.109768} \right)\\ \end{aligned}}  $$

where *F*_*n*_ are climatic control functions and *Θ*_*n*_ (2)–(6) describe an added impact of *n*-th function on plants from different countries of origin.

2 $$ {}{\begin{aligned} \Theta_{0} &=\\ &-3.14988\cdot TH-3.06907\cdot IN-3.7083\cdot AF-3.08391\cdot PK\\  &-3.96199\cdot IR-3.00246\cdot PH+0.641678\cdot BR-2.4291\cdot US \\ &-3.10886\cdot AU-3.23838\cdot Unknown-2.05882\cdot FR\\&\quad+0.635819\cdot KR\\ &+0.851436\cdot TR-2.04774\cdot NG-2.05237\cdot VN\,+\,0.638283\cdot NL\\ &-0.638868\cdot TW-1.01931\cdot KE \end{aligned}}  $$

3 $$ {}{\begin{aligned} \Theta_{1} &=\\ &-3.17194\cdot TH+5.29528\cdot IN\\ &-9.33767\cdot AF+2.71034\cdot PK+8.32015\cdot IR \\ &-5.29786\cdot PH-9.11154\cdot BR-1.99994\cdot US+1.48757\cdot AU\\ &+7.79404\cdot Unknown+2.83045\cdot FR+4.30076\cdot TR-9.17855\cdot NG\\ &-7.81195\cdot VN-8.69424\cdot IQ+2.64235\cdot NL+6.252\cdot TW\\ &-5.80664\cdot MX+3.24466\cdot KE \end{aligned}}  $$

4 $$ {}{\begin{aligned} \Theta_{2} &=\\ &+4.26732\cdot TH-7.58479\cdot IN\\ &-8.24155\cdot AF-1.13634\cdot PK+7.28225\cdot IR+9.38162\cdot PH \\ &+5.94218\cdot BR-3.79285\cdot US+4.46692\cdot AU+1.25946\cdot Unknown\\ &-1.59303\cdot FR-2.44954\cdot KR-6.54573\cdot TR-8.54903\cdot NG\\ &+9.20973\cdot VN+2.88583\cdot IQ+5.73987\cdot NL-9.83768\cdot TW\\ &+4.34869\cdot MX-9.4759\cdot KE \end{aligned}}  $$

5 $$ {}{\begin{aligned} \Theta_{3} &= \\ &+9.29626\cdot IN+1.05261\cdot AF \\ &-1.82252\cdot PK+2.40813\cdot IR+5.6705\cdot PH-5.5971\cdot BR \\ &+5.61499\cdot US+4.50366\cdot Unknown+1.04956\cdot FR\,+\,9.14113\cdot KR \\ &+7.68653\cdot TR-4.25743\cdot NG-6.3334\cdot VN-2.64341\cdot IQ \\ &-5.88418\cdot NL-2.45877\cdot TW+3.098\cdot KE \end{aligned}}  $$

6 $$ {}{\begin{aligned} \Theta_{4} &= \\ &+9.65241\cdot TH \\  &+8.48755\cdot IN+3.99315\cdot AF-7.8144\cdot PK-5.41556\cdot IR \\ &-8.92499\cdot PH-2.06275\cdot BR+6.47815\cdot US+2.7679\cdot AU \\ &-3.88322\cdot FR-4.23494\cdot KR+8.47326\cdot TR+2.2374\cdot NG \\ &+4.6434\cdot VN+7.29815\cdot IQ-5.41947\cdot NL-9.54991\cdot TW \\ &-3.33689\cdot MX-8.30075\cdot KE \end{aligned}}  $$

**Fig. 2:**
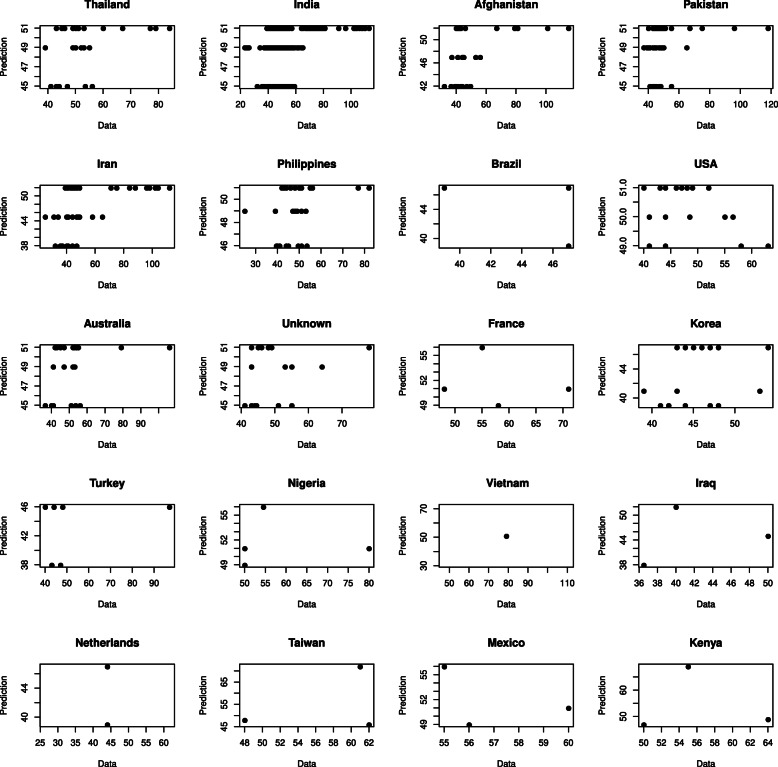
Comparison between actual and predicted days to flowering for plants from different countries of origin. Each dot corresponds to one plant

In formulae (2)–(6) two-letter country codes are used instead of indicator variables *d* for countries of origin: Thailand (TH), India (IN), Afghanistan (AF), Pakistan (PK), Iran (IR), Philippines (PH), Brazil (BR), USA (US), Australia (AU), France (FR), Korea (KR), Turkey (TR), Vietnam (VN), Nigeria (NG), Iraq (IQ), Netherlands (NL), Taiwan (TW), Mexico (MX), Kenya (KE). The interactions between country of origin and environment account for 12% of variation in time to flowering. The comparison between experimental data and model solution for each country of origin is presented in Fig. 2. Though an average error of the model is rather low the range of model solutions is less than that of actual time to flowering in data. This can be due to the fact that the model describes the influence of country of origin on time to flowering and does not account for individual variation of this trait among accessions attributed to one country.

Impacts of climatic factors on time to flowering were estimated by comparison of prediction errors between original model and the one with the factor of interest excluded. It appeared that the impact of day length was 2.3%, precipitation – 8.3%, solar radiation – 5.4%, maximal and minimal temperature – 76.2% and 7.8%, respectively.

To compare the impacts of climatic factors between countries we calculated the Mann-Whitney-Wilcoxon test for mean values of impacts for each pair of countries (Tab. S3). We identified 118 pairs of countries of origin with statistically significant differences between impacts of climatic factors, of which maximal temperature accounts for differences in 109 pairs.

### A model with genotype information

**Fig. 3:**
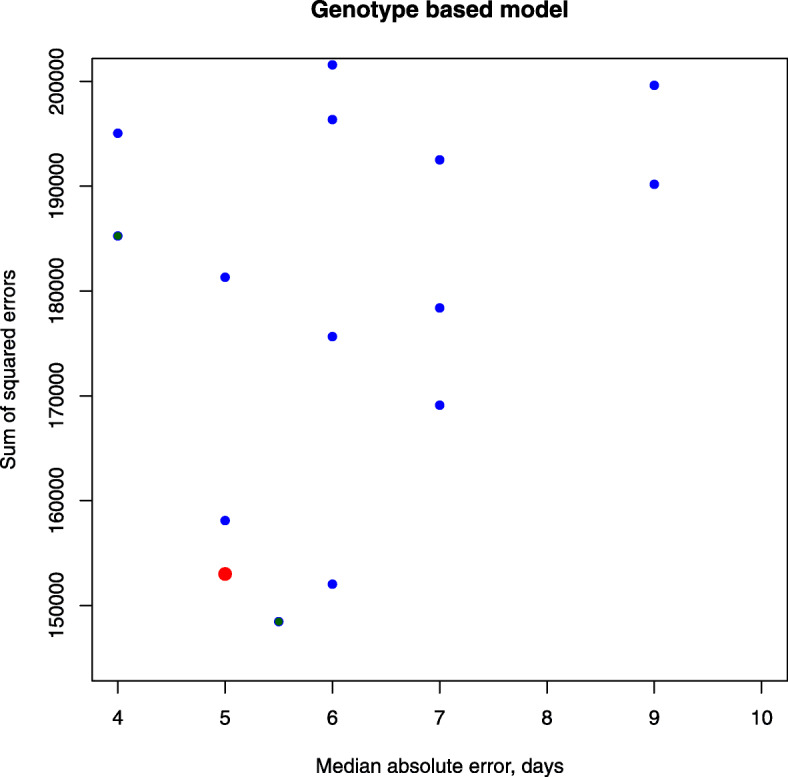
Models with the information about genotype. Each dot is a model and the best model is represented by large red dot

The best model for further investigation was selected as minimizing both the sum of squared differences and median absolute difference in flowering time between model and data. The selected model was obtained with *λ*=150 and defined by (7)–(12) (Fig. 3). The root mean square error in flowering time, resource amount needed to flower, and the coefficient threshold were 11.8 days, *Y*=56.55, and *B*_*min*_=6.283, respectively.

7 $$ {}{\begin{aligned} \Delta y(i,t)&=6.65\cdot F_{0}+7.18\cdot F_{1}+6.22\cdot F_{2}+0.95\cdot F_{3}+0.57\cdot F_{4}\\&+\sum\limits_{n=0}^{4}\Theta_{n} F_{n}\\ F_{0}&= \frac{rain}{(rain - 18.6471)^{2}} \\ F_{1}&=\frac{1}{dl - 0.02289}\\  F_{2}&=\frac{1}{tmin - 1.37714}\\ F_{3}&=\left(\frac{1}{dl - 0.02289} + \frac{1}{tmax - 26.7143} \right) \\ F_{4}&=\frac{1}{rain - 18.6471} \\ \end{aligned}}  $$

where *F*_*n*_ are climatic control functions and *Θ*_*n*_ describe the influence of *n*-th function on plants with different alleles. In (8)–(12) instead of indicator variables *d* from (17) the names of SNP groups are used represented by SNP number and allele combination where ’A’ and ’R’ denote alternative and reference alleles, respectively. The plants with allele combinations included in *Θ*_*n*_ get an additional impact of function *F*_*n*_.

8 $$ {}{\begin{aligned} \Theta_{0} &= \\ &+7.4509\cdot snp4AR-8.16384\cdot snp6RR+7.69515\cdot snp7RR\\ &-7.99183\cdot snp9AR\\ \end{aligned}}  $$

9 $$ {}{\begin{aligned} \Theta_{1} &= \\ &+9.22877\cdot snp1RR+9.28055\cdot snp3AR\\  &+7.15876\cdot snp3RR+8.25876\cdot snp5AA+7.92461\cdot snp5AR\\ &-9.27385\cdot snp6AR+7.54713\cdot snp7AA-6.7873\cdot snp7RR\\ &+6.79649\cdot snp8AR-6.41667\cdot snp8RR+7.17566\cdot snp9RR\\ &+8.43357\cdot snp10AA\\ \end{aligned}}  $$

10 $${}{\begin{aligned} \Theta_{2} &= \\ &+7.37262\cdot snp1AA+9.13604\cdot snp1RR\\ &+9.12136\cdot snp2RR+9.98666\cdot snp3RR+9.22927\cdot snp5AA\\ &-9.23385\cdot snp5AR+9.71658\cdot snp7RR\\ \end{aligned}}  $$

11 $${}{\begin{aligned} \Theta_{3} &= \\ &+9.01623\cdot snp1AA\\ &+8.91822\cdot snp3AA+7.17114\cdot snp3AR\\&\quad+8.60211\cdot snp4AA\\ &+7.46585\cdot snp5AA+7.2088\cdot snp5AR\\&\quad+9.67968\cdot snp5RR\\ &+7.62143\cdot snp6RR-6.30424\cdot snp7AA\\&\quad+8.76958\cdot snp7AR\\ &-9.81541\cdot snp8RR+6.84716\cdot snp10AR-8.12269\cdot snp10RR\\ \end{aligned}}  $$

12 $${}{\begin{aligned} \Theta_{4} &=& \\ &-7.59475\cdot snp1AA-7.87434\cdot snp1AR+6.31816\cdot snp1RR\\  &-7.60708\cdot snp3AR+9.05006\cdot snp3RR+8.54655\cdot snp5RR\\ &+9.38654\cdot snp6RR+8.29729\cdot snp7AA-9.73811\cdot snp7AR\\ &-8.2692\cdot snp9AA-7.24539\cdot snp10AA\\ \end{aligned}}  $$

**Fig. 4:**
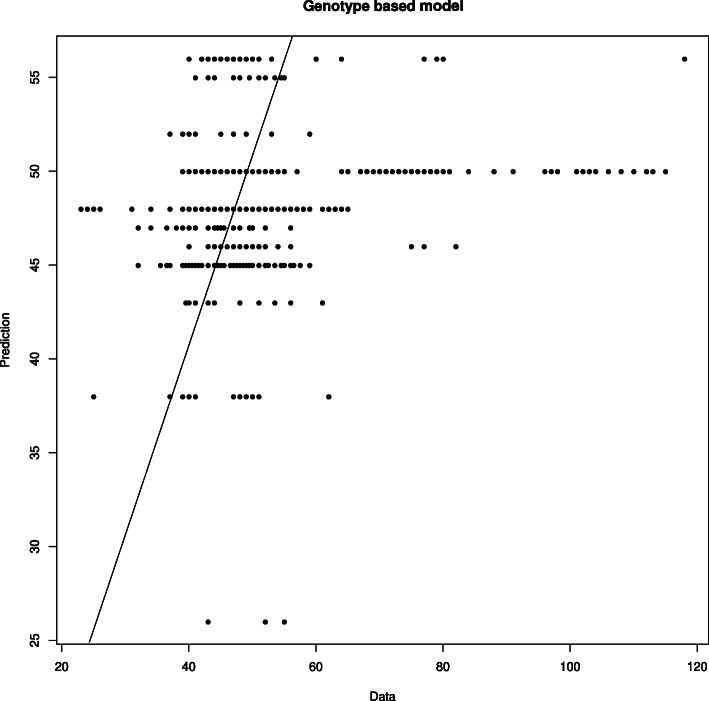
Comparison of days to flowering predicted by the model with genotype information and data. Each dot corresponds to one plant. Straight line corresponds to an ideal case, i.e. when predictions equal data

**Fig. 5:**
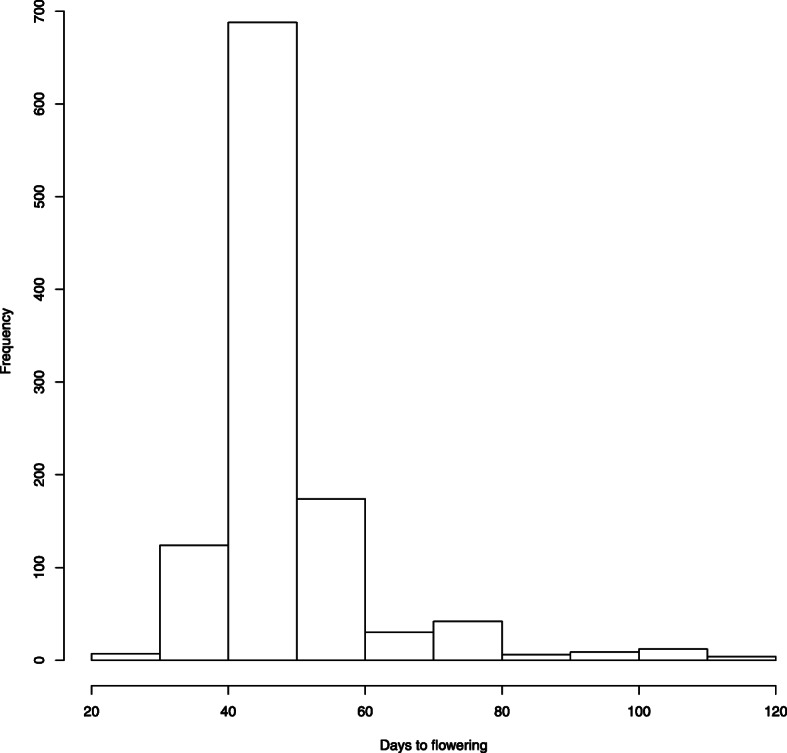
Histogram of times to flowering in the available dataset

The comparison between experimental data and model solution is presented in Fig. 4. Though a model accurately predicts time to flowering for many samples the values over 57 days are underestimated. This can be due to the fact that the samples of this kind make a limited impact on objective function as their frequency in the dataset is low (see the histogram in Fig. 5).

The model ability to generalize to independent datasets was further validated with 4-fold cross-validation test using 75% of samples of the whole dataset for training and 25% of samples for testing. The T-test for comparison of means of root mean square differences between training and test sets showed no statistically significant difference (*P*=0.17>0.05). The root mean square error in flowering time was then compared with Mann-Whitney-Wilcoxon test and again we found no statistically significant differences between training and testing, 28.44 and 30.41 days, respectively.

The genotype-by-environment interactions accounted for 19% of the error in this model according to (20). Impacts of climatic factors on time to flowering were estimated by comparison of prediction errors between original model and the one with the factor of interest excluded. The obtained results showed that the impact of day length was 20%, precipitation – 2.5%, maximal and minimal temperature – 63.5% and 14%, respectively.

To understand how climatic factors affect time to flowering in accessions with different allele combinations the Mann-Whitney-Wilcoxon test was applied to compare mean values of factor impacts for combination pairs. We identified 309 pairs with different response to climatic factors (see Tab. S4). For example, accessions with allele combinations SNP1AA and SNP1AR or SNP1AA and SNP1RR respond differently to day length and minimal temperature, while accessions with allele combinations SNP2AA and SNP2AR, SNP3AA and SNP3AR, SNP4AA and SNP4AR, SNP7AA and SNP7AR, SNP8AA and SNP8AR, SNP9AA and SNP9AR respond differently to all factors.

### Forecasting

Cross-validation performed above on the whole dataset demonstrated that the model with genotype information (17) has good predictive ability. However, for forecasting time to flowering of accessions one needs to be confident that the developed model can generalize to prospective datasets. As the number of datasets available to us was limited, we considered as a “prospective” dataset the dataset from year 2018 while building and validating the model on the rest datasets. The data set for 1984, 1985 and 2016 years was split into validation (105 records) and core datasets as described in Materials and methods. The data from 2018 (292 records) was saved for prospective prediction. The 4-fold cross-validation was then performed for the core dataset that resulted in 100 fitting runs. The T-test for comparison of means of root mean square differences between training and test sets was statistically insignificant (P=0.77>0.05). The Mann-Whitney-Wilcoxon test also showed no statistically significant difference between training and testing, 26.20 and 27.08 days, respectively.

The best model was selected to minimize both the sum of squared differences and median absolute difference between model and data. The root mean square error in the time to flowering prediction was 5.8 days (see Additional file 1). Next this model was applied to predict time to flowering in both validation dataset and “prospective” dataset from 2018 year. We found that the root mean square errors for these datasets were 5.82 and 5.34 days, respectively. The median absolute difference in days to flowering between data and model solution for both validation and “prospective” datasets was also small and equal to 3 and 4 days, respectively. The high accuracy of the model solutions for test, validation and “prospective” datasets demonstrates its good predictive ability and applicability for forecasting time to flowering.

The model (7)-(12) was applied to forecast time to flowering of accessions from the whole dataset (293 accessions) in future years 2020-2030 and in Taiwan. Three replications of daily weather were generated for four RCPs by MarkSim software.

**Fig. 6:**
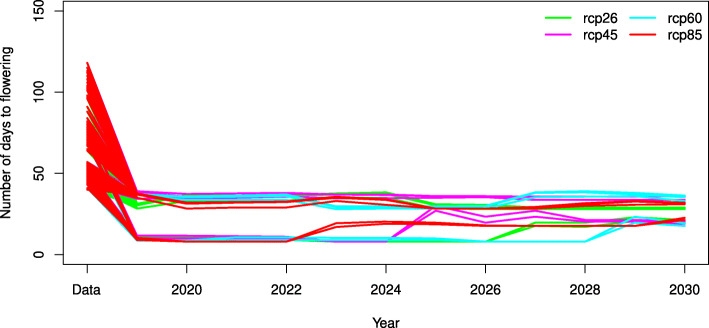
Forecast for time to flowering for 293 accessions averaged over 3 replications of the daily weather

We found that in comparison to available data time to flowering decreases for all accessions in all four climate change scenarios but different groups of accessions follow distinct trajectories that can be combined into two clusters, for which time to flowering is >25 and <25 days, respectively (Fig. 6). Trajectories in the first cluster have small amplitude of fluctuations and flowering time for groups of accessions doesn’t increase significantly in the modeled period. Trajectories attributed to the second cluster increase time to flowering in years 2023, 2025, 2029 and 2027 for rcp85, rcp60, rcp45 and rcp26, respectively. Time to flowering slowly decreases starting from year 2025 for rcp45 for trajectories in this cluster.

**Fig. 7:**
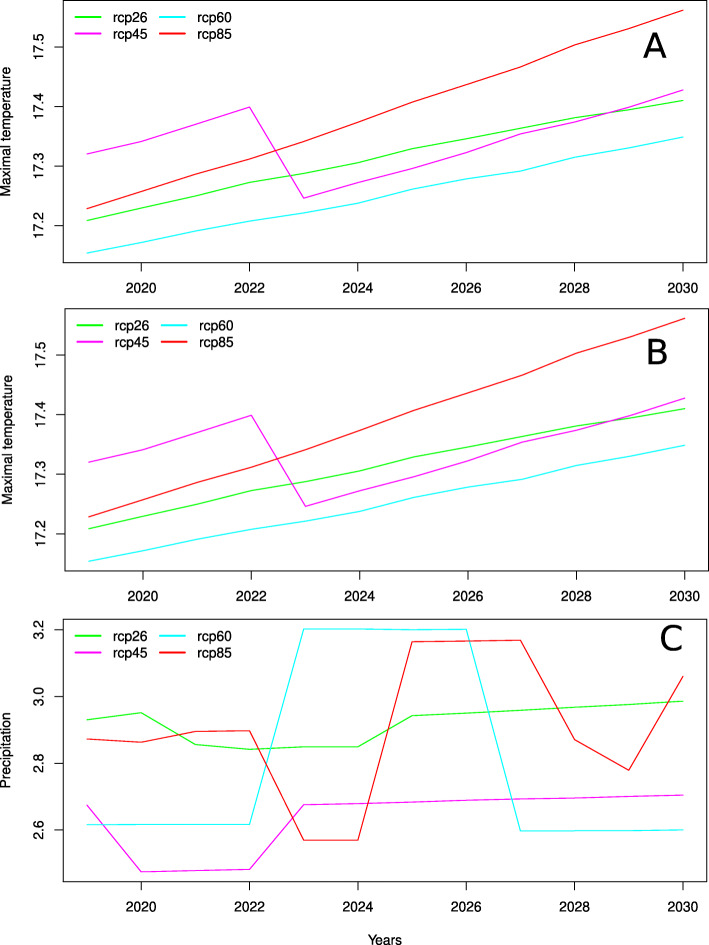
Minimal (**a**) and maximal (**b**) temperature and precipitation (**c**) as predicted by MarkSim and averaged over 3 trials and over the period from sowing to the end of the year

As it is evident from Fig. 7a,b the decrease in time to flowering of all accessions is caused by the prevalent influence of temperature that is steadily growing, except the drop in 2023 for rcp45. Fluctuations of time to flowering can be explained by the fluctuations in precipitation (Fig. 7c). Different trajectories that visualize differences in response between accessions groups to climate change are determined by different influence of climatic factors and maximal temperature in particular on flowering time of accessions with different allele combinations. This conclusion results from the formulae (7)-(12) in which given the same dates of sowing and climate different time to flowering can be obtained only for accessions with different allele combinations. Accessions in the first cluster respond to the climate change almost monotonically and have similar values of time to flowering for the modeled period while accessions that form the second cluster after 2022 increase time to flowering and move closer to the first cluster.

## Discussion

In many tropical and subtropical locations mungbean (*Vigna radiata*) is now an important legume, due to its short duration, quick-cooking seeds, and capacity to fit into warm seasons in crop rotations. Increasingly mungbean is also produced in more temperate regions, such as central Asia, Australia, and the Southern Great plains of the United States. In these settings flowering time is a critical trait, as many rotational partners, such as wheat, grown through the winter often are harvested relatively late in the spring and mungbean production is shifted to conditions, in which short duration is particularly critical to set seed before cool weather begins.

Here we developed new mathematical models that describe dynamic control of time to flowering in mungbean by daily values of maximal and minimal temperature, precipitation, day length and solar radiation. For model development a solution based on combination of Grammatical Evolution and Differential Evolution Entirely Parallel Method was implemented, in which the analytic form of dependencies between predictors (climatic factors, country of origin and genotypes) and phenotype (time to flowering) is automatically inferred by a stochastic optimization technique. This makes it possible to quickly examine different fits to the data and select the optimal one. The models were parameterization on a dataset of 293 mungbean accessions originated from 20 different countries and phenotyped in 4 different environments. GWAS analysis of the data performed recently (Sokolkova et al., in preparation) identified ten polymorphic sites responsible for flowering time.

It was demonstrated in several works that the phenology of adaptive traits, like flowering time, is strongly predicted not only by local environmental factors but also by plant geographic origin [39, 40] (Sokolkova et al., submitted). Besides, as the adaptation to specific environments is blueprinted in genomes [41, 42], different genotypes should respond to local environment in different ways. Our work substantiates and further develops these ideas. We built two types of models that consider the influence of climatic factors on flowering time subject to country of origin or genotype and found that 12% and 19% of variation in time to flowering is respectively accounted for by interactions of climatic factors with these variables. Contrary to previous approaches that measure the combined sensitivity of the phenotype to all environmental factors, our approach makes it possible to identify how specific environmental condition affect this trait. Maximal temperature appeared to be the most critical factor determining how faithfully the model describes the data.

The influence of the country of origin and genotype on plant phenology was further confirmed by applying a comparative approach. We demonstrated that climatic factors differently affect time to flowering in accessions with different allele combinations. For example, accessions with allele combinations SNP1AA and SNP1AR or SNP1AA and SNP1RR respond differently to day length and minimal temperature, while accessions with allele combinations SNP2AA and SNP2AR, SNP3AA and SNP3AR, SNP4AA and SNP4AR, SNP7AA and SNP7AR, SNP8AA and SNP8AR, SNP9AA and SNP9AR respond differently to all factors. Similar conclusion was drawn when considering the interaction of climatic factors with country of origin: we identified 118 country pairs with statistically significant differences between impacts of climatic factors, of which maximal temperature again accounts for differences in 109 pairs.

Forecasting of agronomically important traits is critical to provide timely information for optimum management of growing crops and for national food security. Here we applied the model with genotype information to forecast time to flowering of accessions grown in Taiwan in future years 2020-2030. However to make forecasting reliable one needs to prove that the model can generalize to independent datasets in general and to prospective datasets in particular. Cross-validation performed on the whole dataset demonstrated that our model has good predictive ability: the difference in prediction accuracy on training and test datasets was statistically insignificant. As the number of datasets available to us was limited we considered as a “prospective” dataset the dataset from year 2018 while building and validating the model on the rest datasets. Again, we were able to show that model prediction error on this prospective dataset is small. We conclude that our model has good predictive ability and therefore can be used for forecasting time to flowering.

To forecast time to flowering four climate change scenarios rcp26, rcp45, rcp60 and rcp85 were considered that differently predict Earth radiation balance in 2100 due to possible changes in future anthropogenic emissions of greenhouse gases [38]. We found that while time to flowering decreases for all accessions different groups of accessions respond differently to the same climate change scenarios and that these differences are determined by different influence of climatic factors and maximal temperature in particular on flowering time of accessions with different allele combinations. For example, there is a group of accessions for which time to flowering decreases starting from 2020 to 2022 and then increases at different years for different RCPs. The predicted trends for temperature and precipitation suggests that the fluctuations of flowering time are driven by the fluctuations of precipitation, while the overall decrease in flowering time is due to warming.

## Conclusions

Two types of models that describe dynamic control of time to flowering in mungbean by daily values of maximal and minimal temperature, precipitation, day length and solar radiation subject to country of origin or genotype were considered. The models were used to demonstrate that the phenology of adaptive traits, like flowering time, is strongly predicted not only by local environmental factors but also by plant geographic origin and genotype. Of local environmental factors maximal temperature appeared to be the most critical factor determining how faithfully the model describes the data.

The model with genotype information was cross-validated and applied to forecast time to flowering of accessions grown in Taiwan in future years 2020-2030. Due to the difference in influence of climatic factors on flowering time between accessions with different allele combinations their response to the same climate change scenarios differs. Our results suggest that the overall decrease in flowering time is caused by temperature increase while the fluctuations of flowering time are driven by the fluctuations of precipitation.

## Methods

### Plant material

A mungbean mini core collection comprising 296 genotypes was established from the WorldVeg mungbean collection of 7,965 entries, as described in [4]. Briefly, the whole collection was stratified based on geographical origin of the accessions, then clustered based on eight morphological descriptors. From each cluster 20% of the accessions were randomly chosen, resulting in a core collection of 1,481 entries. The core collection was genotyped with 25 microsatellite markers and the smallest set representing all detected 122 alleles was chosen, resulting in a mini core collection of 296 accessions.

The mini core collection includes accessions from: Thailand (TH), India (IN), Afghanistan (AF), Pakistan (PK), Iran (IR), Philippines (PH), Brazil (BR), USA (US), Australia (AU), France (FR), Korea (KR), Turkey (TR), Vietnam (VN), Nigeria (NG), Iraq (IQ), Netherlands (NL), Taiwan (TW), Mexico (MX), Kenya (KE). Table S1 shows the number of samples from each region.

The available dataset was composed during field experiments:

- 1984: sown on 28/08/1984; harvest on 24/10/1984. Geographical coordinates: N 23 ^∘^ 6’ 50" E 120 ^∘^ 17’ 55",
- 1985: sown on 17/09/1985; harvest on 03/10/1985. Geographical coordinates: N 23 ^∘^ 6’ 50" E 120 ^∘^ 17’ 55",
- 2016: sown on 16/06/2016, harvest from 22/08 to mid-September. Geographical coordinates: N 17 ^∘^ 30’ 28" E 78 ^∘^ 16’ 10",
- 2018: sown 21/09/2018 and harvested from Dec. 24-28 2018. Geographical coordinates: N 23 ^∘^ 6’ 50" E 120 ^∘^ 17’ 55".

A histogram of times to flowering in the dataset is presented in Fig. 5.

### GWAS

We used 10 SNPs found in a recent GWAS analysis associated with flowering time (Sokolkova et al., in review). In brief, the SNPs were identified in 293 lines based on 5041 SNPs, using a single-locus linear mixed model, implemented in FaST-LMM toolset (Factored Spectrally Transformed Linear Mixed Models) [23]. The LMM model was implemented with the first ten PCA axes scores used as covariates. We used a false discovery rate (FDR) [24] of 0.05 to determine significant trait associated loci. In this GWAS analysis, ten SNPs were identified associated with flowering time. Coordinates, allele combinations and additional information for these SNPs are presented in Tab. S2.

### Climate data

The data on climatic conditions for each day in the period of field experiments were taken from publicly available site https://rp5.ru/Weather_in_the_world(Website provides weather forecasts for 172’500 locations, as well as observational data, reported from weather stations) and NASA [25] (These data were obtained from the NASA Langley Research Center (LaRC) POWER Project funded through the NASA Earth Science/Applied Science Program):

1. *dl* or *D* is day length,
2. *tmin* or *Tn* is a minimal temperature,
3. *tmax* or *Tx* is a maximal temperature,
4. *rain* or *P* is precipitation,
5. *srad* or *S* is a solar radiation.

### Simulation model

In [18] the framework was developed for combining weather and SNP data. We further enhance the method by introducing non-linearity and automatic function selection. The simulation model describes how the readiness to flower *y*(*i*,*t*) of a plant *i* increases at on each day *t* from sowing (*t*=0) to actual flowering (*t*=*T*_*i*_). To increase its readiness the plant accumulates resources and the daily increment *Δy*(*i*,*t*) depends both on the environment at day *t* and plant-environment interaction.

13 $$ \begin{aligned} y(i,t)&=y(i,t-1)+H(\Delta y(i,t))\Delta y(i,t)\\y(i,0)&=0\quad y(i,T_{i})\geq Y\quad y(i,T_{i}-1)< Y \end{aligned}  $$

where *Y* is a resource amount that a plant needs to accumulate to be able to flower and *H*() is a Heaviside function.

Several forms of dependence have been proposed in the literature. Here we propose a more general approach in which the readiness to flower is determined automatically in analytic form using Grammatical Evolution (GE) [26, 27]. In GE, the analytic function form is built by decoding the sequence called “word” of *W* integers called codons. Decoding is performed according to simple rules of substitution that establish a correspondence between codons and either an elementary arithmetic operation: ‘+‘, ‘-‘, ‘*‘, ‘/‘, or expression: X, (X - Const), 1/(X - Const), where X is a name of a predictor and Const is some constant number.

The daily increment of readiness to flower depends on climatic factors (14).

14 $$ {}\Delta y(i,t)=\sum_{n=0}^{N-1}\beta_{n}\cdot F_{n}(D_{i}(t),Tn_{i}(t),Tx_{i}(t),P_{i}(t),S_{i}(t))+\varepsilon_{i}   $$

where $\beta _{n},\;n=0,\dots,N-1$ are coefficients, $F_{n},\;n=0,\dots,N-1$ are non-linear control functions and *D*,*Tn*,*Tx*,*P*,*S* are climatic factors, combined in a vector *X*(*t*)=(*D*_*i*_(*t*),*Tn*_*i*_(*t*),*Tx*_*i*_(*t*),*P*_*i*_(*t*),*S*_*i*_(*t*)).

To study the adaptation to environment at the country of origin we represent these countries as *L*=20 binary variables, where $l=1,\dots,L$ enumerates countries: Thailand, India, Afghanistan, Pakistan, Iran, Philippines, Brazil, USA, Australia, France, Korea, Turkey, Nigeria, Vietnam, Iraq, Netherlands, Taiwan, Mexico, Kenya. 20 samples were labeled ’Unknown’ as the source country information is unavailable, however we kept these accessions as they possess unique alleles. For each plant enumerated with $i=0,\dots,I-1$ one of the *L* variables $d_{i}^{l}$ takes the value ’1’ to indicate collection site and others are ’0’. The interaction between control function and country of origin is modeled with additional term in (15) that has the form of a weighted sum of *N*·*L* pairwise products of control functions *F*_*n*_ and each binary site variable $d_{i}^{l}$.

A model then takes the form:

15 $$ {}\begin{aligned} \Delta y(i,t)&=\sum_{n=0}^{N-1}\beta_{n}\cdot F_{n}(X)+\sum_{n=0}^{N-1}\sum_{l=1}^{L}H(\left\vert\zeta_{l\cdot N+n}\right\vert-B_{min})\\&\quad\cdot\zeta_{l\cdot N+n}\cdot F_{n}(X)\cdot d^{l}_{i}+\varepsilon_{i}  \end{aligned}  $$

where in addition to notations used in (13) and (14) new coefficients *ζ*_*l*·*N*+*n*_ define the influence of climatic control function *F*_*n*_ on phenotype of plants originated from location *l* so that condition *ζ*_*l*·*N*+*n*_≠0 points on plant adaptation to environment at the country of origin. To make estimation of new coefficients more reliable we introduced the threshold parameter *B*_*min*_ and set all coefficients ≤*B*_*min*_ to be zero.

Available genetic information (Tab. S2) defines groups of samples with different allele combinations. We denote *K* number of SNP and *J*=3 combinations of alternative (ALT) and reference (REF) alleles ALT/ALT, ALT/REF and REF/REF by 0, 1, and 2, respectively. Then to include GWAS results into the model we define *J*·*K* groups of plants so that members of the same group have the same combination of alleles in one of the SNP positions. Thus we define a matrix *D* with the number of rows equal to the number of plants *I* and *J*·*K* columns. Then, the elements of matrix *D* are defined by (16). Thus, the form of the regression function adapts to allele combination of a plant by changing the weights of control functions.

16 $$\begin{array}{@{}rcl@{}} d^{3k+j}_{i} & \,=\,\left\{\begin{array}{ll} 1 & \quad \text{if in plant}\ i \text{ the combination for SNP} k \text{ is}\ j\\ 0 & \quad \text{otherwise}\vspace*{10pt} \end{array}\right. \end{array} $$

A model then takes the form:

17 $$ \begin{aligned} \Delta y(i,t)&=\sum_{n=0}^{N-1}\beta_{n}\cdot F_{n}(X)\\&\quad+\sum_{n=0}^{N-1}\sum_{k=0}^{K-1}\sum_{j=0}^{J-1}H(\left\vert\rho_{(3k+j)N+n}\right\vert-B_{min})\\&\quad\cdot\rho_{(3k+j)N+n}\cdot F_{n}(X)\cdot d^{3k+j}_{i}+\varepsilon_{i}  \end{aligned}  $$

where in addition to notations used in (13) and (14), new coefficients *ρ*_(3*k*+*j*)*N*+*n*_ define the effect of genotype-by-climatic factor interaction. *B*_*min*_ is a threshold parameter for coefficients *ρ*_(3*k*+*j*)*N*+*n*_.

The vector of unknown model parameters *θ*∈*R*^*r*^ consist of *W* codons, coefficients $\beta _{n},\;n=0,\dots,N-1$, *ζ*_*l*·*N*+*n*_ (or *ρ*_(3*k*+*j*)*N*+*n*_) together with threshold parameter *B*_*min*_ and resource amount *Y*.

In this work we implemented approach, in which the analytic form of climatic control functions together with unknown model parameters were inferred automatically by stochastic minimization of the deviation of the model output from data, formulae (18) and (19) for models with country of origin (15) and genotype (17), respectively.

The number of parameters *r* for *W*=60 codons of *N*=5 climatic control functions and *L*=20 countries of origin was *r*=172 for the model with country of origin information (15). The number of parameters for model (17) with *K*=10 SNPs and the same number of codons was 222.

18 $$\begin{array}{@{}rcl@{}} Q(\theta)&=&\sum_{i=0}^{I-1}(T_{i} - \tau_{i}(\theta))^{2}  \\  &+&\lambda\left\{\sum_{n=0}^{N-1}\left\vert\beta_{n}\right\vert+\sum_{n=0}^{N-1}\sum_{l=1}^{L}\left\vert\zeta_{l\cdot N+n}\right\vert\right\} \\ &+&\alpha\left\{\sum_{n=0}^{N-1}\beta_{n}^{2}+\sum_{n=0}^{N-1}\sum_{l=1}^{L}\zeta_{l\cdot N+n}^{2}\right\}  \end{array} $$

19 $$\begin{array}{@{}rcl@{}} {}Q(\theta)&=&\sum_{i=0}^{I-1}(T_{i} - \tau_{i}(\theta))^{2}  \\  &+&\lambda\left\{\sum_{n=0}^{N-1}\left\vert\beta_{n}\right\vert+\sum_{n=0}^{N-1}\sum_{k=0}^{K-1}\sum_{j=0}^{J-1}\left\vert\rho_{(3k+j)N+n}\right\vert\right\} \\ &+&\alpha\left\{\sum_{n=0}^{N-1}\beta_{n}^{2}+\sum_{n=0}^{N-1}\sum_{k=0}^{K-1}\sum_{j=0}^{J-1}\rho_{(3k+j)N+n}^{2}\right\}  \end{array} $$

In both formulae (18) and (19) *α*=1 and *λ* are regularization parameters, *τ*_*i*_(*θ*) is a number of days from planting to flowering predicted by a model with parameters *θ*.

We used a combination of GE and Differential Evolution Entirely Parallel (DEEP) Method [28–30] to fit model to available data. Differential Evolution was proposed by Storn and Price in 1995 [31] as a heuristic stochastic optimization method. DEEP was developed by us for application in the field of bioinformatics [28]. It includes several recently proposed enhancements [29, 32].

### Estimation of impacts of interactions and climatic factors to the model

The impact of either genotype-by-environment or country of origin-by-environment interactions in the models defined by parameter vector *θ* was calculated as the percentage of error increase in the model with information of interest removed (20).

20 $$ S=100\%\cdot\frac{\left(Q(\theta^{0})-\sum_{i=0}^{I-1}(T_{i} - \tau_{i}(\theta))^{2}\right)}{Q(\theta^{0})}   $$

where *θ*^0^=*θ* except all *ρ*_∗_=0 or *ζ*_∗_=0.

Impacts of climatic factors on time to flowering were estimated by comparison of prediction errors between original model and the one with the factor of interest excluded. The difference in mean values of impacts of climatic factors between different countries of origin or genotypes was compared with the Mann-Whitney-Wilcoxon test.

### Cross-validation of the model with genotype information and forecasts

We used the model with genotype information to forecast time to flowering in accessions grown in Taiwan in 2020-2030. However, for forecasting time to flowering in future years one needs to estimate how the models will generalize to independent datasets in general and to prospective datasets in particular. Available data allows us to simulate such a setup.

Firstly, the 4-fold cross-validation of the model was performed on the whole dataset using 75% of samples for training and 25% of samples for testing. Secondly, to test the model ability to predict time to flowering in prospective datasets we saved 20% of all records (105) from years 1984, 1985 and 2016 as the validation set and referred to the rest of 80% accessions as the core set.

We used four-fold cross-validation to build a model with genotype information on the core set. The core set was split 25 times randomly into training and test sets containing 75% and 25% of accessions correspondingly. The model was fitted to the training set and the accuracy of prediction was estimated on a test set as a root mean square error in time to flowering between predictions and data. Next the best model with the smallest prediction error was selected and tested on the validation set and after that was used to predict time to flowering for 2018 data (292 records). The insignificant difference in mean square errors in time to flowering for training and test sets in cross-validation runs and high prediction accuracy of flowering time in both validation and 2018 dataset ensures that the model was not overfitted and can be generalized to an independent prospective datasets.

### Synthetic weather generation

We forecasted time to flowering for accessions grown in Taiwan. The daily weather forecasts for Taiwan from 2020 to 2030 were produced using the weather generator program MarkSim. MarkSim was designed to simulate weather from known sources of monthly climate data [33–37] and takes into account the socio-economic development scenarios described by the four representative carbon dioxide concentration profiles (RCPs) adopted by the Intergovernmental Panel on Climate Change (IPCC) in the fifth assessment report (AR5) in 2014. The profiles correspond to a wide range of possible changes in future anthropogenic emissions of greenhouse gases and are called rcp26, rcp45, rcp60 and rcp85 in accordance with the possible violation values for radiation earth balance in 2100 in respect to the preindustrial epoch (+2.6, +4.5, +6.0 and +8.5 *W*/*m*^2^, respectively) [38].

## Supplementary information

**Additional file 1** Supporting information. Additional file 1 contains information on SNP based groups, climatic data for these groups, details on Grammatical evolution method.

## Data Availability

The datasets and programs used and/or analyzed during the current study are available from the corresponding author on request. List of entries and phenotypic data of The World Vegetable Center mungbean mini core collection can be accessed at https://static-content.springer.com/esm/art%3A10.1186%2Fs12864-015-1556-7/MediaObjects/12864_2015_1556_MOESM1_ ESM.xlsx, passports are available at http://seed.worldveg.org/search/passport.

## Abbreviations

APSIM: Agricultural production systems sIMulator
SNP: Single nucleotide polymorphism
NASA: National aeronautics and space administration
GWAS: Genome-wide association studies
FaST-LMM: Factored spectrally transformed linear mixed models
PCA: Principal component analysis
FDR: False discovery rate
RCP: Representative carbon dioxide concentration profile
IPCC: Intergovernmental panel on climate change
AR: Assessment report
GE: Grammatical evolution
DEEP: Differential evolution entirely parallel
ANOVA: Analysis of variance
TTF: Time to flowering
