# Supplementary material for: Dynamical climatic model for time to flowering in Vigna radiata
Source: BMC Plant Biol. 2020 Oct 14;20(Suppl 1):202. doi: 10.1186/s12870-020-02408-1 (PMC7556928; doi:10.1186/s12870-020-02408-1)
Supplement: Supplementary file 1 — Additional file 1 Supporting information. Additional file 1 contains information on SNP based groups, climatic data for these groups, details on Grammatical evolution method. [file 12870_2020_2408_MOESM1_ESM.pdf]

Dynamical climatic model for time to flowering in Vigna radiata. Supporting Information

K.Kozlov, A.Sokolkova, C.Lee, C.Ting, R.Schafleitner,
E. Bishop-von Wettberg, S.Nuzhdin and M.Samsonova

August 24, 2019

S1 Dataset

Table S1: Number of samples collected at each country.

#	Name	Number
1	Thailand	44
2	India	699
3	Afghanistan	45
4	Pakistan	82
5	Iran	75
6	Philippines	36
7	Brazil	3
8	USA	18
9	Australia	25
10	Unknown	20
11	France	4
12	Korea	20
13	Turkey	6
14	Nigeria	4
15	Vietnam	1
16	Iraq	3
17	Netherlands	2
18	Taiwan	3
19	Mexico	3
20	Kenya	3

Table S2: Coordinates, allele combinations and additional information for ten SNPs identified in GWAS

Genotype	SNP1	SNP2	SNP3	SNP4	SNP5	SNP6	SNP7	SNP8	SNP9	SNP10
CHROM	1	1	1	1	3	4	9	9	9	208
POS	20062538	25659766	25684221	28003044	6153449	17585806	20243588	20243588	20243588	47000
ID	6197526024	6197494040	6197281049	620561807	6188196014	6200069040	6208281033	6208281034	6208281035	6191369025
REF	G	G	G	A	A	C	C	C	G	G
ALT	C	A	A	G	G	T	A	T	A	T
QUAL	.	.	.	.	.	.	.	.	.	.
FILTER	PASS	PASS	PASS	PASS	PASS	PASS	PASS	PASS	PASS	PASS
INFO	.	.	.	.	.	.	.	.	.	.
FORMAT	GT	GT	GT	GT	GT	GT	GT	GT	GT	GT
VI000020 AY	0/0	1/0	0/0	./.	0/0	0/0	1/1	./.	./.	0/0
VI001509 AG	0/0	0/0	0/0	0/0	0/0	0/0	0/0	0/0	0/0	0/0
VI001557 BG	0/0	0/0	0/0	0/0	0/0	0/0	0/0	0/0	0/0	0/0
VI001652 BG	0/0	0/0	0/0	0/0	0/0	0/0	0/0	0/0	0/0	0/0
VI000212 A BLM	0/0	0/0	0/0	0/0	0/0	0/0	0/0	0/0	0/0	0/0
VI000461 BG	0/0	0/0	0/0	0/0	1/1	0/0	0/0	0/0	0/0	0/0
VI000578 AG	0/0	0/0	0/0	0/0	0/0	0/0	0/0	0/0	0/0	0/0
VI000735 BG	0/0	0/0	0/0	0/0	0/0	0/0	0/0	0/0	0/0	0/0
VI000852 AG	1/1	1/1	1/1	1/1	1/1	0/1	1/1	1/1	1/1	0/1
VI001124 AG	1/1	1/0	1/0	0/1	1/1	0/1	1/0	0/1	1/0	0/1
VI001282 AG	0/0	0/0	0/0	0/0	0/1	0/0	0/0	0/0	0/0	0/0
VI001411 AG	0/0	0/0	0/0	0/0	0/1	0/0	0/0	0/0	0/0	0/0
VI000099 AG	0/0	0/0	0/0	0/0	0/1	0/0	0/0	0/0	0/0	0/0
VI001514 AG	0/0	0/0	0/0	0/0	0/0	0/0	0/0	0/0	0/0	0/0
VI001562 AG	0/0	./.	0/0	0/0	0/0	0/0	0/0	0/0	0/0	0/0
VI001654 BG	0/0	0/0	0/0	0/0	0/0	0/0	0/0	0/0	0/0	0/0
VI000232 AG	0/1	1/0	1/0	0/1	1/1	0/1	1/0	0/1	1/0	0/1
VI000470 AG	0/0	0/0	0/0	0/0	0/1	0/0	0/0	0/0	0/0	0/0
VI000589 B BR	0/1	0/0	0/0	0/0	0/1	0/0	0/0	0/0	0/0	0/0

VI000736 AG	1/1	0/0	0/0	0/0	1/1	0/0	./.	./.	./.	0/0
VI000938 AG	0/0	0/0	0/0	0/0	0/0	0/0	0/0	0/0	0/0	0/0
VI001126 BG	0/0	0/0	0/0	0/0	0/0	0/0	0/0	0/0	0/0	0/0
VI001284 AG	0/0	0/0	0/0	0/0	0/1	0/0	0/0	0/0	0/0	0/0
VI001412 AG	0/0	1/0	0/0	0/0	0/0	0/0	0/0	0/0	0/0	0/0
VI000105 BG	0/0	0/0	0/0	0/0	1/1	0/0	0/0	0/0	0/0	0/0
VI001520 A BLM	0/0	0/0	0/0	0/0	0/0	0/0	0/0	0/0	0/0	0/0
VI001576 BG	0/0	0/0	0/0	0/0	0/0	0/0	./.	./.	./.	0/0
VI001678 BG	0/0	0/0	0/0	0/0	0/0	0/0	0/0	0/0	0/0	0/0
VI000238 AG	0/0	0/0	0/0	0/0	0/0	0/0	0/0	0/0	0/0	0/0
VI000532 BG	0/0	0/0	0/0	0/0	0/0	0/0	0/0	0/0	0/0	0/0
VI000616 BG	0/0	0/0	0/0	0/0	0/1	0/0	0/0	0/0	0/0	0/0
VI000749 AG	0/0	0/0	0/0	0/0	0/0	0/0	0/0	0/0	0/0	0/0
VI000942 AG	1/1	1/0	1/1	1/1	1/1	0/1	1/1	1/1	1/1	0/1
VI001162 AG	0/0	0/0	0/0	0/0	1/1	0/0	0/0	0/0	0/0	0/0
VI001339 AG	0/0	0/0	0/0	0/0	1/1	0/0	0/0	0/0	0/0	0/0
VI001419 BG	0/0	0/0	0/0	0/0	0/0	0/0	0/0	0/0	0/0	0/0
VI000164 BG	0/0	0/0	0/0	0/0	0/0	0/0	0/0	0/0	0/0	0/0
VI001533 BG	0/0	0/0	0/0	0/0	0/0	0/0	./.	./.	./.	0/0
VI001579 BG	0/0	0/0	0/0	0/0	0/0	0/0	./.	./.	./.	./.
VI001692 AG	0/0	0/0	0/0	0/0	0/0	0/0	0/0	0/0	0/0	0/0
VI000253 AG	0/0	0/0	0/0	0/0	0/0	0/0	0/0	0/0	0/0	0/0
VI000537 BG	0/0	0/0	0/0	0/0	0/1	0/0	0/0	0/0	0/0	0/0
VI000618 AG	0/0	0/0	0/0	0/0	0/1	0/0	0/0	0/0	0/0	0/0
VI000764 AG	0/0	0/0	0/0	0/0	1/1	0/0	0/0	0/0	0/0	./.
VI000953 AG	1/1	1/1	1/1	1/1	0/0	0/1	1/1	1/1	1/1	0/1
VI001191 BG	0/0	0/0	0/0	0/0	1/1	0/0	0/0	0/0	0/0	./.
VI001385 AG	0/0	0/0	0/0	0/0	0/0	0/0	0/0	0/0	0/0	0/0
VI001435 AG	0/0	0/0	0/0	0/0	0/0	0/0	0/0	0/0	0/0	0/0
VI000170 B BR	0/0	0/0	0/0	0/0	0/1	0/0	0/0	0/0	0/0	0/0
VI001535 BG	0/0	0/0	0/0	0/0	0/0	0/0	0/0	0/0	0/0	0/0
VI001605 BG	0/0	0/0	0/0	0/0	0/1	0/0	0/0	0/0	0/0	0/0
VI001698 BG	0/0	0/0	0/0	0/0	0/0	0/0	1/0	0/0	0/0	0/0
VI000316 AG	0/0	0/0	0/0	0/0	0/0	0/0	0/0	0/0	0/0	0/0
VI000542 BY	0/0	0/0	0/0	./.	0/0	0/0	./.	./.	./.	0/0
VI000625 B BR	0/0	0/0	0/0	0/0	0/0	0/0	0/0	0/0	0/0	0/0
VI000766 BG	0/0	0/0	0/0	0/0	1/1	0/0	0/0	0/0	0/0	0/0
VI000981 BG	0/0	0/0	0/0	0/0	0/0	0/0	0/0	0/0	0/0	0/0
VI001211 AG	0/1	1/0	1/0	1/1	0/1	0/1	1/1	1/1	1/1	0/0
VI001400 AG	0/1	1/0	1/0	0/1	0/0	0/0	1/0	0/1	1/0	0/0
VI001448 A BLM	0/0	0/0	0/0	0/0	0/1	0/0	0/0	0/0	0/0	0/0
VI000175 BY	0/0	0/0	0/0	./.	1/1	0/0	0/0	0/0	0/0	0/0
VI001539 AG	0/0	0/0	0/0	0/0	0/0	0/0	0/0	0/0	0/0	0/0
VI001612 AG	0/0	0/0	0/0	0/0	0/1	0/0	0/0	0/0	0/0	./.
VI001728 AG	0/0	0/0	0/0	0/0	0/0	0/0	0/0	0/0	0/0	0/0
VI000317 BG	0/1	1/0	1/0	0/1	0/1	0/1	1/0	0/1	1/0	0/0
VI000551 AG	0/0	0/0	0/0	0/0	0/0	0/0	0/0	0/0	0/0	0/0
VI000680 AG	0/0	0/0	0/0	0/0	0/1	0/0	0/0	0/0	0/0	0/0
VI000805 BG	0/0	0/0	0/0	0/0	1/1	0/0	0/0	0/0	0/0	0/0
VI001023 BG	0/0	0/0	0/0	0/0	1/1	0/0	0/0	0/0	0/0	0/0
VI001221 AG	1/1	1/1	1/1	1/1	0/0	0/1	1/1	1/1	1/1	0/1
VI001403 BR	0/0	0/0	0/0	0/0	0/0	0/0	./.	./.	./.	0/0
VI001471 AG	0/0	0/0	0/0	0/0	0/0	0/0	0/0	0/0	0/0	0/0
VI000188 A BLM	0/0	0/0	0/0	0/0	0/0	0/0	0/0	0/0	0/0	0/0
VI001548 AG	0/0	0/0	0/0	0/0	0/0	0/0	0/0	0/0	0/0	0/0
VI001628 AG	0/0	0/0	0/0	0/0	0/1	0/0	0/0	0/0	0/0	0/0
VI000319 AG	1/1	1/1	1/1	1/1	0/0	0/0	1/1	1/1	1/1	0/1
VI000554 AG	0/0	0/0	0/0	0/0	0/0	0/0	0/0	0/0	0/0	0/0
VI000723 AG	0/1	0/0	0/0	0/0	0/0	0/0	0/0	0/0	0/0	0/0
VI000815 BG	0/0	0/0	0/0	0/0	0/0	0/0	./.	./.	./.	0/0
VI001066 BG	0/0	0/0	0/0	./.	0/0	0/0	./.	./.	./.	0/0
VI001244 AG	1/1	1/1	1/1	1/1	0/0	0/1	1/1	1/1	1/1	0/1
VI001406 BG	0/0	0/0	0/0	0/0	0/0	0/0	0/0	0/0	0/0	0/0
VI001482 BG	0/0	0/0	0/0	0/0	0/0	0/0	0/0	0/0	0/0	0/0
VI000203 B BR	0/0	0/0	0/0	0/0	0/0	0/0	0/0	0/0	0/0	0/0
VI001556 BG	0/0	0/0	0/0	0/0	0/0	0/0	0/0	0/0	0/0	0/0
VI001651 BG	0/0	0/0	0/0	0/0	0/0	0/0	0/0	0/0	0/0	0/0
VI000380 AG	1/1	1/1	1/1	1/1	1/1	0/1	1/1	1/1	1/1	0/1
VI000559 AG	0/0	0/0	0/0	0/0	0/1	0/0	0/0	0/0	0/0	0/0
VI000732 AG	0/1	1/0	1/0	0/1	0/0	0/1	1/0	0/1	1/0	0/1
VI000818 BG	0/0	0/0	0/0	0/0	1/1	0/0	0/0	0/0	0/0	0/0
VI001096 AG	0/0	0/0	0/0	0/0	1/1	0/0	0/0	0/0	0/0	0/0
VI001268 BG	0/1	1/0	1/0	0/1	1/1	0/1	1/0	0/1	1/0	0/1
VI001408 BG	0/0	0/0	0/0	0/0	0/0	0/0	0/0	0/0	0/0	0/0
VI001490 AG	1/1	0/0	0/0	0/0	0/0	0/0	0/0	0/0	0/0	0/0
VI001733 BG	0/0	0/0	0/0	0/0	./.	0/0	0/0	0/0	0/0	0/0
VI003235 AG	0/0	0/0	0/0	0/0	0/0	0/0	0/0	0/0	0/0	0/0
VI003332 AG	0/1	0/0	0/0	0/0	0/1	0/0	0/0	0/0	0/0	0/0
VI003455 AG	0/0	0/0	0/0	0/0	0/0	0/0	0/0	0/0	0/0	./.
VI001974 BG	0/0	0/0	0/0	./.	1/1	0/0	./.	./.	./.	0/0
VI002176 AG	0/0	0/0	0/0	0/0	0/0	0/0	0/0	0/0	0/0	0/0
VI002284 BG	0/1	1/0	1/0	0/1	0/0	0/1	1/0	0/1	1/0	0/1
VI002672 AG	0/0	0/0	0/0	0/0	0/0	0/0	0/0	0/0	0/0	0/0
VI002926 AG	0/0	0/0	0/0	0/0	0/0	0/0	0/0	0/0	0/0	0/0
VI003035 AG	0/0	0/0	0/0	0/0	0/0	0/0	0/0	0/0	0/0	0/0
VI003159 AG	./.	0/0	0/0	0/0	0/0	0/0	0/0	0/0	0/0	./.
VI001743 BG	0/0	0/0	0/0	0/0	0/0	0/0	0/0	0/0	0/0	0/0
VI003242 AG	0/0	0/0	0/0	0/0	1/1	0/0	0/0	0/0	0/0	0/0
VI003337 BG	0/0	0/0	0/0	0/0	0/0	0/0	0/0	0/0	0/0	0/0
VI003456 AG	0/0	0/0	0/0	./.	0/0	0/0	0/0	0/0	0/0	0/0
VI001993 BG	0/0	0/0	0/0	0/0	1/1	0/0	0/0	0/0	0/0	0/0
VI002176 BG	0/0	0/0	0/0	0/0	0/0	0/0	0/0	0/0	0/0	0/0
VI002402 BG	0/0	0/0	0/0	0/0	0/0	0/0	0/0	0/0	0/0	0/0
VI002532 AG	0/0	0/0	0/0	0/0	0/0	0/0	0/0	0/0	0/0	0/0
VI002739 AG	1/1	1/1	1/1	1/1	0/0	0/1	1/1	1/1	1/1	0/1
VI002934 AG	0/0	0/0	0/0	0/0	0/0	0/0	0/0	0/0	0/0	./.
VI003057 BG	0/0	0/0	0/0	0/0	0/0	0/0	0/0	0/0	0/0	0/0
VI003172 BG	0/0	0/0	0/0	0/0	0/0	0/0	0/0	0/0	0/0	0/0
VI001756 BG	0/0	0/0	0/0	0/0	1/1	0/0	0/0	0/0	0/0	0/0
VI003251 A BL	./.	0/0	0/0	0/0	0/0	0/0	0/0	0/0	0/0	0/0
VI003364 AG	0/0	0/0	0/0	0/0	0/1	0/0	0/0	0/0	0/0	0/0
VI003465 BG	0/0	0/0	0/0	0/0	0/0	0/0	0/0	0/0	0/0	0/0
VI002009 BG	0/0	0/0	0/0	0/0	1/1	0/0	0/0	0/0	0/0	0/0
VI002190 BG	1/1	1/1	1/1	1/1	1/1	0/1	1/1	1/1	1/1	0/1
VI002432 AG	0/1	1/0	1/0	0/1	0/0	0/0	1/0	0/1	1/0	0/0
VI002537 AG	0/0	0/0	0/0	0/0	0/1	0/0	0/0	0/0	0/0	0/0
VI002802 A BR	0/0	0/0	0/0	0/0	0/0	0/0	0/0	0/0	0/0	0/0
VI002986 AG	0/0	0/0	0/0	0/0	0/0	0/0	0/0	0/0	0/0	0/0
VI003062 BG	0/0	0/0	0/0	0/0	0/0	0/0	0/0	0/0	0/0	./.
VI003181 B GM	0/0	0/0	0/0	0/0	0/0	0/0	0/0	0/0	0/0	0/0

VI001762 A GM	0/0	0/0	0/0	0/0	1/1	0/0	0/0	0/0	0/0	0/0
VI003251 A BLM	./.	0/0	0/0	0/0	0/0	0/0	0/0	0/0	0/0	0/0
VI003379 BG	0/0	0/0	0/0	0/0	0/1	0/0	0/0	0/0	0/0	./.
VI003470 BG	0/0	0/0	0/0	0/0	0/0	0/0	0/0	0/0	0/0	0/0
VI002012 BG	0/0	0/0	0/0	0/0	0/0	0/0	./.	./.	./.	0/0
VI002195 AG	1/1	1/1	1/1	1/1	./.	0/1	1/1	1/1	1/1	0/1
VI002437 BG	0/0	0/0	0/0	0/0	./.	0/0	0/0	0/0	0/0	0/0
VI002569 BG	0/0	0/0	0/0	./.	0/0	0/0	./.	./.	./.	0/0
VI002859 BG	0/0	0/0	0/0	0/0	1/1	0/0	0/0	0/0	0/0	0/0
VI002993 BG	0/0	0/0	0/0	./.	./.	0/0	0/0	0/0	0/0	0/0
VI003068 A BR	./.	0/0	0/0	./.	0/0	0/0	./.	./.	./.	./.
VI003183 AG	0/0	0/0	0/0	0/0	0/0	0/0	0/0	0/0	0/0	0/0
VI001806 AG	0/0	0/0	0/0	0/0	0/1	0/0	0/0	0/0	0/0	0/0
VI003252 BG	0/0	0/0	0/0	./.	0/0	0/0	0/0	0/0	0/0	0/0
VI003382 BG	0/0	0/0	0/0	0/0	0/0	0/0	0/0	0/0	0/0	0/0
VI003480 BG	0/0	0/0	0/0	./.	0/0	0/0	0/0	0/0	0/0	0/0
VI002051 BG	0/0	0/0	0/0	0/0	0/1	0/0	0/0	0/0	0/0	0/0
VI002197 BG	0/0	0/0	0/0	./.	1/1	0/0	0/0	0/0	0/0	0/0
VI002456 AG	0/0	1/0	1/0	0/1	1/1	0/0	1/0	0/1	1/0	./.
VI002587 AG	0/0	0/0	0/0	0/0	1/1	0/0	0/0	0/0	0/0	0/0
VI002860 AG	0/0	0/0	0/0	0/0	0/1	0/0	0/0	0/0	0/0	0/0
VI002999 AG	0/0	0/0	0/0	0/0	1/1	0/0	0/0	0/0	0/0	0/0
VI003070 AG	0/0	0/0	0/0	0/0	0/0	0/0	0/0	0/0	0/0	0/0
VI003187 BG	0/0	0/0	0/0	./.	1/1	0/0	./.	./.	./.	0/0
VI001806 BG	0/0	0/0	0/0	0/0	1/1	0/0	0/0	0/0	0/0	0/0
VI003255 AG	0/0	0/0	0/0	0/0	0/0	0/0	0/0	0/0	0/0	./.
VI003407 AG	0/0	0/0	0/0	0/0	0/0	0/0	0/0	0/0	0/0	0/0
VI003490 AG	0/0	0/0	0/0	0/0	0/0	0/0	0/0	0/0	0/0	0/0
VI002063 BG	0/0	0/0	0/0	0/0	1/1	0/0	0/0	0/0	0/0	./.
VI002206 AG	0/1	1/0	1/0	0/1	1/1	0/1	1/0	0/1	1/0	1/1
VI002469 AG	0/0	0/0	0/0	0/0	0/0	0/0	0/0	0/0	0/0	0/0
VI002611 AG	0/0	0/0	0/0	0/0	0/0	0/0	0/0	0/0	0/0	0/0
VI002872 BG	0/0	0/0	0/0	0/0	1/1	0/0	0/0	0/0	0/0	0/0
VI003019 A BLM	./.	0/0	0/0	0/0	0/0	0/0	0/0	0/0	0/0	0/0
VI003083 BG	0/0	0/0	0/0	0/0	1/1	0/0	0/0	0/0	0/0	0/0
VI003212 B BLM	./.	0/0	0/0	0/0	0/0	0/0	0/0	0/0	0/0	0/0
VI001820 BG	0/0	0/0	0/0	0/0	0/0	0/0	0/0	0/0	0/0	./.
VI003276 BG	0/0	0/0	0/0	0/0	0/0	0/0	0/0	0/0	0/0	0/0
VI003413 BG	0/0	0/0	0/0	0/0	0/0	0/0	0/0	0/0	0/0	0/0
VI002173 AG	0/0	0/0	0/0	0/0	0/1	0/0	0/0	0/0	0/0	0/0
VI002239 AG	0/0	0/0	0/0	0/0	0/0	0/0	0/0	0/0	0/0	0/0
VI002487 AG	0/0	0/0	0/0	0/0	./.	0/0	0/0	0/0	0/0	0/0
VI002646 AG	0/0	0/0	0/0	0/0	0/0	0/0	0/0	0/0	0/0	0/0
VI002877 BG	0/0	0/0	0/0	0/0	0/1	0/0	0/0	0/0	0/0	0/0
VI003019 BG	0/0	0/0	0/0	0/0	0/1	0/0	0/0	0/0	0/0	0/0
VI003114 AG	0/0	0/0	0/0	0/0	0/0	0/0	0/0	0/0	0/0	0/0
VI003220 AG	0/0	0/0	0/0	./.	0/0	0/0	0/0	0/0	0/0	0/0
VI001859 BG	0/0	0/0	0/0	0/0	0/1	0/0	0/0	0/0	0/0	0/0
VI003329 AG	0/0	0/0	0/0	0/0	0/0	0/0	0/0	0/0	0/0	0/0
VI003440 AG	0/0	0/0	0/0	0/0	0/0	0/0	0/0	0/0	0/0	0/0
VI002173 BG	0/0	0/0	0/0	0/0	0/0	0/0	0/0	0/0	0/0	0/0
VI002523 AG	0/1	1/0	1/0	0/1	0/0	0/0	1/0	0/1	1/0	0/0
VI002647 AG	1/1	1/1	1/1	1/1	0/0	0/1	1/1	1/1	1/1	0/1
VI002894 B BR	./.	0/0	0/0	0/0	0/0	0/0	0/0	0/0	0/0	0/0
VI003034 BG	1/1	1/0	1/0	0/1	0/0	0/1	1/0	0/1	1/0	0/1
VI003135 B BL	./.	0/0	0/0	./.	0/0	0/0	./.	./.	./.	0/0
VI003232 AG	./.	0/0	0/0	0/0	0/0	0/0	0/0	0/0	0/0	0/0
VI003493 BG	0/0	0/0	0/0	0/0	0/0	0/0	0/0	0/0	0/0	0/0
VI004639 AG	0/0	0/0	0/0	0/0	0/1	0/0	0/0	0/0	0/0	0/0
VI004810 BG	0/0	0/0	0/0	./.	0/0	0/0	0/0	0/0	0/0	0/0
VI004915 BG	0/0	0/0	0/0	0/0	0/0	0/0	0/0	0/0	0/0	0/0
VI003563 A BR	0/0	0/0	0/0	0/0	0/0	0/0	0/0	0/0	0/0	0/0
VI003685 AG	0/0	0/0	0/0	0/0	0/0	0/0	0/0	0/0	0/0	0/0
VI003755 BG	0/0	0/0	0/0	0/0	0/1	0/0	0/0	0/0	0/0	0/0
VI003893 AG	0/0	0/0	0/0	0/0	0/0	0/0	0/0	0/0	0/0	0/0
VI003944 B BR	0/0	0/0	0/0	0/0	1/1	0/0	0/0	0/0	0/0	0/0
VI004006 A GM	0/0	0/0	0/0	0/0	1/1	0/0	0/0	0/0	0/0	0/0
VI004096 BG	0/0	0/0	0/0	0/0	0/0	0/0	0/0	0/0	0/0	0/0
VI004302 AG	0/0	0/0	0/0	0/0	0/0	0/0	0/0	0/0	0/0	0/0
VI003514 BG	0/0	0/0	0/0	0/0	0/0	0/0	0/0	0/0	0/0	0/0
VI004666 AG	0/0	0/0	0/0	0/0	0/0	0/0	0/0	0/0	0/0	0/0
VI004811 BG	0/0	0/0	0/0	0/0	0/0	0/0	0/0	0/0	0/0	0/0
VI004931 AG	0/0	0/0	0/0	0/0	0/0	0/0	0/0	0/0	0/0	0/0
VI003577 AG	0/0	0/0	0/0	0/0	0/0	0/0	0/0	0/0	0/0	0/0
VI003699 B BG	0/0	0/0	0/0	0/0	0/0	0/0	0/0	0/0	0/0	0/0
VI003760 BG	0/0	0/0	0/0	0/0	0/0	0/0	0/0	0/0	0/0	0/0
VI003894 B BLM	0/0	0/0	0/0	0/0	0/0	0/0	0/0	0/0	0/0	0/0
VI003947 B BR	0/0	0/0	0/0	0/0	0/0	0/0	0/0	0/0	0/0	0/0
VI004010 AG	1/1	1/1	1/1	1/1	1/1	0/1	1/1	1/1	1/1	0/1
VI004129 A BLM	0/0	0/0	0/0	0/0	0/0	0/0	0/0	0/0	0/0	0/0
VI004307 AG	0/0	0/0	0/0	0/0	0/0	0/0	0/0	0/0	0/0	0/0
VI003517 BG	0/0	0/0	0/0	0/0	0/0	0/0	0/0	0/0	0/0	0/0
VI004691 AG	0/0	0/0	0/0	0/0	0/0	0/0	0/0	0/0	0/0	0/0
VI004822 BG	0/0	0/0	0/0	0/0	0/1	0/0	0/0	0/0	0/0	0/0
VI004933 AG	0/0	0/0	0/0	0/0	0/0	0/0	0/0	0/0	0/0	0/0
VI003602 AG	0/0	0/0	0/0	0/0	0/1	0/0	0/0	0/0	0/0	0/0
VI003720 BG	0/0	0/0	0/0	0/0	0/0	0/0	0/0	0/0	0/0	0/0
VI003785 BG	0/0	0/0	0/0	0/0	0/0	0/0	0/0	0/0	0/0	0/0
VI003907 AG	0/0	0/0	0/0	0/0	0/0	0/0	0/0	0/0	0/0	0/0
VI003948 B BR	0/0	0/0	0/0	0/0	0/1	0/0	0/0	0/0	0/0	0/0
VI004024 AG	0/0	0/0	0/0	0/0	0/0	0/0	0/0	0/0	0/0	0/0
VI004138 BG	0/0	0/0	0/0	0/0	1/1	0/0	0/0	0/0	0/0	0/0
VI004312 AG	0/0	0/0	0/0	0/0	1/1	0/0	0/0	0/0	0/0	0/0
VI003534 AG	0/0	0/0	0/0	0/0	0/1	0/0	0/0	0/0	0/0	0/0
VI004694 BG	0/0	0/0	0/0	0/0	0/1	0/0	0/0	0/0	0/0	0/0
VI004838 AG	0/0	0/0	0/0	0/0	0/1	0/0	0/0	0/0	0/0	0/0
VI004934 AG	0/0	0/0	0/0	0/0	0/0	0/0	0/0	0/0	0/0	0/0
VI003642 AG	0/0	0/0	0/0	0/0	0/0	0/0	0/0	0/0	0/0	0/0
VI003725 BG	0/0	0/0	0/0	0/0	0/0	0/0	0/0	0/0	0/0	0/0
VI003795 AG	0/1	1/0	1/0	0/1	0/0	0/1	1/0	0/1	1/0	0/1
VI003914 AG	0/0	0/0	0/0	0/0	0/0	0/0	0/0	0/0	0/0	0/0
VI003951 AG	0/0	0/0	0/0	0/0	1/1	0/0	0/0	0/0	0/0	0/0
VI004044 BG	0/0	0/0	0/0	0/0	0/1	0/0	0/0	0/0	0/0	0/0
VI004145 B BLM	0/0	0/0	0/0	0/0	0/0	0/0	0/0	0/0	0/0	0/0
VI004347 B BLM	0/0	0/0	0/0	0/0	0/0	0/0	0/0	0/0	0/0	0/0
VI003534 BG	0/0	0/0	0/0	0/0	0/0	0/0	0/0	0/0	0/0	0/0
VI004710 AG	0/0	0/0	0/0	0/0	0/0	0/0	0/0	0/0	0/0	0/0
VI004842 AG	0/0	0/0	0/0	0/0	0/0	0/0	0/0	0/0	0/0	0/0
VI004937 AG	0/0	0/0	0/0	0/0	0/0	0/0	0/0	0/0	0/0	0/0
VI003648 BG	0/0	0/0	0/0	0/0	0/0	0/0	0/0	0/0	0/0	0/0

VI003733 BG	0/0	0/0	0/0	0/0	0/0	0/0	0/0	0/0	0/0	0/0
VI003801 BG	0/0	0/0	0/0	0/0	0/1	0/0	0/0	0/0	0/0	0/0
VI003925 B BLM	0/1	0/0	0/0	0/0	0/0	0/0	0/0	0/0	0/0	0/0
VI003954 BG	0/0	0/0	0/0	0/0	0/0	0/0	0/0	0/0	0/0	0/0
VI004045 A DGM	0/0	0/0	0/0	0/0	0/1	0/0	0/0	0/0	0/0	0/0
VI004184 AG	0/0	0/0	0/0	0/0	0/0	0/0	0/0	0/0	0/0	0/0
VI004351 AG	0/0	0/0	0/0	0/0	0/0	0/0	0/0	0/0	0/0	0/0
VI003548 AG	0/0	0/0	0/0	0/0	0/0	0/0	0/0	0/0	0/0	0/0
VI004734 AG	0/0	0/0	0/0	0/0	0/0	0/0	0/0	0/0	0/0	0/0
VI004853 BG	0/0	0/0	0/0	0/0	0/1	0/0	0/0	0/0	0/0	0/0
VI004942 BG	0/0	0/0	0/0	0/0	0/0	0/0	0/0	0/0	0/0	0/0
VI003658 BG	0/0	0/0	0/0	0/0	0/0	0/0	0/0	0/0	0/0	0/0
VI003734 B BR	0/0	0/0	0/0	./.	0/0	0/0	0/0	0/0	0/0	0/0
VI003882 A BLM	0/0	0/0	0/0	0/0	0/1	0/0	0/0	0/0	0/0	0/0
VI003927 AG	0/0	0/0	0/0	0/0	0/0	0/0	0/0	0/0	0/0	0/0
VI003957 AG	0/0	0/0	0/0	0/0	0/0	0/0	0/0	0/0	0/0	0/0
VI004048 A DGM	0/0	0/0	0/0	0/0	0/0	0/0	0/0	0/0	0/0	0/0
VI004243 B BR	0/0	0/0	0/0	0/0	0/0	0/0	0/0	0/0	0/0	0/0
VI004423 AG	0/0	0/0	0/0	0/0	0/0	0/0	0/0	0/0	0/0	0/0
VI003554 AG	0/0	0/0	0/0	0/0	0/0	0/0	0/0	0/0	0/0	0/0
VI004743 AG	0/0	0/0	0/0	0/0	0/0	0/0	0/0	0/0	0/0	0/0
VI004871 BG	0/0	0/0	0/0	0/0	0/1	0/0	0/0	0/0	0/0	0/0
VI003664 AG	0/0	0/0	0/0	0/0	0/0	0/0	0/0	0/0	0/0	0/0
VI003734 B DG	0/0	0/0	0/0	0/0	0/0	0/0	0/0	0/0	0/0	0/0
VI003886 B BR	0/0	0/0	0/0	0/0	0/1	0/0	0/0	0/0	0/0	0/0
VI003929 A BL	0/0	0/0	0/0	0/0	0/0	0/0	0/0	0/0	0/0	0/0
VI003958 B BLM	0/0	0/0	0/0	0/0	0/1	0/0	0/0	0/0	0/0	0/0
VI004069 BG	0/0	0/0	0/0	0/0	0/1	0/0	0/0	0/0	0/0	0/0
VI004244 B BR	0/0	0/0	0/0	0/0	0/0	0/0	0/0	0/0	0/0	0/0
VI004432 B BR	0/0	0/0	0/0	0/0	0/0	0/0	0/0	0/0	0/0	0/0
VI003560 BG	0/0	0/0	0/0	0/0	0/0	0/0	0/0	0/0	0/0	0/0
VI004789 BG	0/0	0/0	0/0	0/0	0/1	0/0	0/0	0/0	0/0	0/0
VI004877 AG	0/0	0/0	0/0	0/0	1/1	0/0	0/0	0/0	0/0	0/0
VI003678 BG	0/0	0/0	0/0	0/0	0/0	0/0	0/0	0/0	0/0	0/0
VI003744 AG	0/0	0/0	0/0	0/0	0/0	0/0	0/0	0/0	0/0	0/0
VI003886 BY	0/0	0/0	0/0	./.	1/1	0/0	0/0	0/0	0/0	0/0
VI003942 AG	0/0	0/0	0/0	0/0	0/1	0/0	0/0	0/0	0/0	0/0
VI003959 BG	0/0	0/0	0/0	0/0	0/1	0/0	0/0	0/0	0/0	0/0
VI004096 AG	0/0	./.	0/0	0/0	0/0	0/0	0/0	0/0	0/0	0/0
VI004297 AG	0/0	0/0	0/0	0/0	0/0	0/0	0/0	0/0	0/0	0/0
VI004480 AG	0/0	0/0	0/0	0/0	0/0	0/0	0/0	0/0	0/0	0/0
VI004954 BG	0/1	1/0	1/0	0/1	0/1	0/1	1/0	0/1	1/0	0/1
VI005022 BG	0/0	0/0	0/0	./.	0/0	0/0	0/0	0/0	0/0	0/0
VI004956 AG	0/0	0/0	0/0	0/0	0/0	0/0	0/0	0/0	0/0	0/0
VI004957 AG	0/0	0/0	0/0	0/0	0/0	0/0	1/0	0/0	0/0	0/0
VI005030 BY	0/0	0/0	0/0	./.	1/1	0/0	./.	./.	./.	0/0
VI004958 BG	0/1	1/0	1/0	0/1	0/0	0/1	1/0	0/1	1/0	0/0
VI005041 AG	0/0	0/0	0/0	0/0	1/1	0/0	0/0	0/0	0/0	0/0
VI004965 BG	0/0	0/0	0/0	0/0	0/0	0/0	0/0	0/0	0/0	0/0
VI005066 A GM	0/0	0/0	0/0	0/0	0/0	0/0	0/0	0/0	0/0	0/0
VI004968 AG	0/0	0/0	0/0	0/0	0/0	0/0	0/0	0/0	0/0	0/0
VI014178 BG	0/0	0/0	0/0	0/0	0/0	0/0	./.	./.	./.	0/0
VI004969 AG	0/1	0/0	0/0	0/0	0/0	0/0	0/0	0/0	0/0	0/0
VI004973 B BLM	0/0	0/0	0/0	0/0	0/0	0/0	0/0	0/0	0/0	0/0

S2 Comparison of impacts between countries of origin

Table S3: The p-values of Mann-Witney-Wilcoxon test for impacts comparison between locations. Statistically significant (< 0.05) results are marked with '*'.

Country	Country	dl	rain	tmax	tmin	srad
Thailand	India	(-) 0.08231878	(*) 0.009177681	(-) 0.6113828	(*) 1.254299e-05	(*) 0.01265307
Thailand	Afghanistan	(*) 0.04135227	(-) 0.1751676	(*) 0.001991005	(-) 0.07505439	(-) 0.6277654
Thailand	Pakistan	(*) 4.632101e-05	(*) 0.001313911	(-) 0.2831065	(*) 2.703927e-05	(*) 0.005101518
Thailand	Iran	(-) 0.07026715	(*) 0.02390502	(*) 0.0005135332	(-) 0.2843073	(-) 0.6158324
Thailand	Philippines	(-) 0.4912285	(-) 0.07965556	(-) 0.6702042	(*) 0.01783887	(-) 0.2889161
Thailand	Brazil	(*) 0.02740394	(-) 0.1324723	(*) 0.004301685	(*) 0.01523903	(-) 0.2951083
Thailand	USA	(-) 0.4838502	(*) 0.01943626	(*) 0.001212865	(*) 0.0001743974	(*) 8.281441e-05
Thailand	France	(-) 0.3031214	(-) 0.8517848	(*) 0.02157449	(-) 0.3991929	(-) 0.3038872
Thailand	Korea	(*) 0.002094048	(*) 0.01880827	(*) 1.860692e-10	(*) 0.04516127	(-) 0.3382041
Thailand	Turkey	(*) 0.001220531	(-) 0.9048021	(*) 0.001180581	(*) 0.02086678	(-) 0.2492817
Thailand	Nigeria	(-) 0.613252	(-) 0.7226192	(*) 0.018588	(-) 0.8366958	(-) 0.5877349
Thailand	Vt.icj	(-) 0.2858776	(-) 0.4437206	(*) 0.0159671	(-) 0.7500863	(-) 0.4659651
Thailand	Netherlands	(-) 0.06210855	(-) 0.0794681	(*) 0.01891764	(-) 0.2670666	(-) 0.6462285
Thailand	Taiwan	(-) 0.484639	(-) 0.08909167	(*) 0.004930229	(-) 0.0695976	(-) 0.1163054
Thailand	Mexico	(-) 0.2858776	(-) 0.4437206	(*) 0.0159671	(-) 0.7500863	(-) 0.4659651
Thailand	Kenya	(-) 0.3939606	(-) 0.3375123	(*) 0.007344918	(-) 0.8440056	(-) 0.3262961
India	Afghanistan	(-) 0.05801452	(-) 0.8340702	(*) 3.918165e-05	(*) 0.01308507	(-) 0.2981449
India	Pakistan	(*) 2.309735e-07	(-) 0.1424585	(-) 0.2728643	(-) 0.2544444	(-) 0.1730949
India	Iran	(-) 0.6130181	(-) 0.4189734	(*) 3.847663e-08	(*) 6.513788e-09	(-) 0.1172553
India	Brazil	(*) 0.01111509	(-) 0.3399996	(*) 0.003119042	(-) 0.118117	(-) 0.2928778
India	USA	(*) 0.04272306	(-) 0.1440786	(*) 0.0001172729	(*) 0.02413217	(*) 3.926485e-05
India	Unknown	(-) 0.3742756	(-) 0.5325217	(-) 0.1017169	(*) 0.02933369	(-) 0.3851038
India	France	(-) 0.07586325	(-) 0.6065361	(*) 0.02530953	(-) 0.7507128	(-) 0.435886
India	Korea	(*) 1.240026e-05	(-) 0.05903492	(*) 4.817399e-14	(-) 0.3097981	(-) 0.4856499
India	Turkey	(*) 0.0001919668	(-) 0.7906568	(*) 0.0005466348	(-) 0.1942231	(-) 0.4672017
India	Nigeria	(-) 0.3147981	(-) 0.7484842	(*) 0.02604613	(-) 0.7932659	(-) 0.6768331
India	Vt.icj	(-) 0.1094445	(-) 0.1541823	(*) 0.02276097	(-) 0.4530354	(-) 0.7449781
India	Netherlands	(*) 0.03554443	(-) 0.1109934	(*) 0.01580696	(-) 0.492531	(-) 0.8157945
India	Taiwan	(-) 0.3735967	(-) 0.1819773	(*) 0.003613561	(-) 0.1478923	(-) 0.1294462
India	Mexico	(-) 0.1094445	(-) 0.1541823	(*) 0.02276097	(-) 0.4530354	(-) 0.7449781
India	Kenya	(-) 0.09391791	(-) 0.546056	(*) 0.004550542	(-) 0.9385152	(-) 0.3123115
Afghanistan	Pakistan	(-) 0.1875013	(-) 0.3549388	(*) 0.0009358674	(*) 0.004919364	(-) 0.1755535
Afghanistan	Iran	(-) 0.2765727	(-) 0.443397	(-) 0.4922128	(*) 0.02372929	(-) 0.7322214
Afghanistan	Philippines	(*) 0.03716316	(-) 0.6442585	(*) 0.005023318	(-) 0.2035224	(-) 0.7176009
Afghanistan	Brazil	(*) 0.0289353	(-) 0.3690586	(*) 0.006281662	(-) 0.07608364	(-) 0.3473317
Afghanistan	USA	(*) 0.01952693	(-) 0.147152	(*) 0.0003060414	(*) 0.00253517	(*) 0.0004439586
Afghanistan	Australia	(-) 0.1363871	(-) 0.8875908	(*) 0.01850223	(-) 0.71248	(-) 0.3414764
Afghanistan	Unknown	(-) 0.9829142	(-) 0.7920173	(*) 0.03459309	(-) 0.7428305	(-) 0.9772676
Afghanistan	France	(*) 0.03324776	(-) 0.741387	(*) 0.01915433	(-) 0.4979138	(-) 0.2800181
Afghanistan	Korea	(*) 0.000318086	(-) 0.07356949	(*) 8.521858e-10	(-) 0.1853578	(-) 0.5641496
Afghanistan	Turkey	(*) 0.001297945	(-) 0.9415296	(*) 0.0002800662	(-) 0.09749421	(-) 0.3792772
Afghanistan	Nigeria	(-) 0.2870223	(-) 0.7551108	(*) 0.02011728	(-) 0.8546399	(-) 0.7005596
Afghanistan	Vt.icj	(-) 0.0779625	(-) 0.2793881	(*) 0.01823782	(-) 0.5577413	(-) 0.7695723

Afghanistan	Netherlands	(-) 0.06737233	(-) 0.1450636	(*) 0.02470863	(-) 0.4122109	(-) 0.8531939
Afghanistan	Taiwan	(-) 0.4277907	(-) 0.2143665	(*) 0.007145068	(-) 0.1186726	(-) 0.07277047
Afghanistan	Mexico	(-) 0.0779625	(-) 0.2793881	(*) 0.01823782	(-) 0.5577413	(-) 0.7695723
Afghanistan	Kenya	(-) 0.06243589	(-) 0.5072703	(*) 0.01040981	(-) 0.9829466	(-) 0.3473317
Pakistan	Iran	(*) 0.002066602	(-) 0.9155347	(*) 6.109881e-05	(*) 1.380329e-06	(-) 0.07649112
Pakistan	Philippines	(*) 8.750501e-05	(-) 0.507548	(-) 0.1266371	(-) 0.4952932	(-) 0.1931755
Pakistan	Brazil	(*) 0.008137841	(-) 0.5025232	(*) 0.004345071	(-) 0.1357802	(-) 0.3328665
Pakistan	USA	(*) 0.0001157346	(-) 0.3282689	(*) 0.0001079712	(-) 0.09367573	(*) 0.000230147
Pakistan	Australia	(*) 0.002485652	(-) 0.3444054	(-) 0.7179568	(-) 0.08311469	(-) 0.5208004
Pakistan	Unknown	(-) 0.2427489	(-) 0.2034814	(-) 0.1979383	(*) 0.008544828	(-) 0.1321802
Pakistan	France	(*) 0.006297336	(-) 0.4331588	(*) 0.01915618	(-) 0.7893576	(-) 0.543687
Pakistan	Korea	(*) 9.537164e-07	(-) 0.1267565	(*) 1.37879e-11	(-) 0.3915493	(-) 0.6913034
Pakistan	Turkey	(*) 0.0001756447	(-) 0.6958661	(*) 0.0009096036	(-) 0.242137	(-) 0.6007314
Pakistan	Nigeria	(-) 0.1123305	(-) 0.4898373	(*) 0.01969172	(-) 0.7657277	(-) 0.8128705
Pakistan	Vt.icj	(*) 0.01819548	(-) 0.09296554	(*) 0.01915732	(-) 0.4470756	(-) 0.8691919
Pakistan	Netherlands	(*) 0.02898802	(-) 0.1286348	(*) 0.01941071	(-) 0.5663846	(-) 0.9061951
Pakistan	Taiwan	(-) 0.2129772	(-) 0.356586	(*) 0.004345071	(-) 0.1626537	(-) 0.1548386
Pakistan	Mexico	(*) 0.01819548	(-) 0.09296554	(*) 0.01915732	(-) 0.4470756	(-) 0.8691919
Pakistan	Kenya	(*) 0.01409889	(-) 0.7284461	(*) 0.005043731	(-) 0.9809588	(-) 0.3328665
Iran	Philippines	(-) 0.3573699	(-) 0.8165104	(*) 0.001841505	(*) 0.001181619	(-) 0.6383574
Iran	Brazil	(*) 0.03080813	(-) 0.3666033	(*) 0.007047313	(*) 0.0240653	(-) 0.2981347
Iran	USA	(*) 0.03700416	(-) 0.3006711	(*) 0.0002508818	(*) 7.663941e-05	(*) 8.839396e-05
Iran	Australia	(-) 0.3329986	(-) 0.4975359	(*) 0.004999203	(*) 0.03246758	(-) 0.3394351
Iran	Unknown	(-) 0.493087	(-) 0.3097502	(*) 0.02612057	(-) 0.1436239	(-) 0.9016189
Iran	France	(-) 0.1119809	(-) 0.5746335	(*) 0.02555155	(-) 0.1309634	(-) 0.2268872
Iran	Korea	(*) 0.0005933287	(-) 0.163894	(*) 2.677011e-10	(*) 0.01057437	(-) 0.2640954
Iran	Turkey	(*) 0.001185414	(-) 0.9444721	(*) 0.0003844481	(*) 0.01411655	(-) 0.3002663
Iran	Nigeria	(-) 0.4690479	(-) 0.6043809	(*) 0.03637258	(-) 0.4436393	(-) 0.4965951
Iran	Vt.icj	(-) 0.1356551	(-) 0.2526112	(*) 0.02700661	(-) 0.8040361	(-) 0.6273176
Iran	Netherlands	(-) 0.07022901	(-) 0.2278041	(*) 0.02719421	(-) 0.1789934	(-) 0.7223658
Iran	Taiwan	(-) 0.4587542	(-) 0.3218188	(*) 0.01135298	(*) 0.04360437	(-) 0.06538206
Iran	Mexico	(-) 0.1356551	(-) 0.2526112	(*) 0.02700661	(-) 0.8040361	(-) 0.6273176
Iran	Kenya	(-) 0.08753127	(-) 0.6516818	(*) 0.01219067	(-) 0.340822	(-) 0.3282155
Philippines	Brazil	(*) 0.01303161	(-) 0.3421213	(*) 0.004772763	(-) 0.1195093	(-) 0.3031158
Philippines	USA	(-) 0.2014687	(-) 0.4082816	(*) 0.001642896	(-) 0.05975352	(*) 0.0005014189
Philippines	France	(-) 0.10858	(-) 0.6194587	(*) 0.04711591	(-) 0.7180437	(-) 0.4035936
Philippines	Korea	(*) 0.0005078572	(-) 0.2409851	(*) 8.948527e-10	(-) 0.3084117	(-) 0.4410514
Philippines	Turkey	(*) 0.0004464712	(-) 0.7872631	(*) 0.001285943	(-) 0.1950878	(-) 0.3585639
Philippines	Vt.icj	(-) 0.1679758	(-) 0.214448	(*) 0.04006943	(-) 0.6517422	(-) 0.6845049
Philippines	Netherlands	(*) 0.03898853	(-) 0.1795123	(*) 0.02019303	(-) 0.413254	(-) 0.7433669
Philippines	Taiwan	(-) 0.5090528	(-) 0.3158543	(*) 0.006597843	(-) 0.1620227	(-) 0.1394144
Philippines	Mexico	(-) 0.1679758	(-) 0.214448	(*) 0.04006943	(-) 0.6517422	(-) 0.6845049
Philippines	Kenya	(-) 0.1320542	(-) 0.5976604	(*) 0.006597843	(-) 0.9789518	(-) 0.355649
Brazil	USA	(*) 0.01023304	(-) 0.6502274	(*) 0.007677153	(-) 0.6150729	(-) 0.3924872
Brazil	Australia	(*) 0.02094637	(-) 0.2978597	(*) 0.005956376	(-) 0.05319321	(-) 0.3155152
Brazil	Unknown	(*) 0.03929347	(-) 0.2512406	(*) 0.00698333	(-) 0.06077968	(-) 0.3603594
USA	Australia	(-) 0.305789	(-) 0.2517926	(*) 0.003252842	(*) 0.01472202	(*) 0.007241607
USA	Unknown	(-) 0.08124469	(-) 0.1167515	(*) 0.0024658	(*) 0.004533149	(*) 0.0009459807
USA	Korea	(*) 0.001822977	(-) 0.7808181	(*) 1.992004e-07	(-) 0.5780944	(-) 0.9068204
USA	Turkey	(*) 0.0004020684	(-) 0.7637782	(*) 0.006897299	(-) 1	(-) 0.8153843
USA	Iraq	(-) 0.249403	(-) 0.898109	(*) 0.02981458	(-) 0.08847917	(-) 0.05524409
USA	Netherlands	(*) 0.02712024	(-) 0.3762018	(*) 0.02729425	(-) 1	(-) 1
USA	Taiwan	(-) 0.5451855	(-) 0.364307	(*) 0.0137111	(-) 0.5134425	(-) 0.2085644
USA	Kenya	(-) 0.5788134	(-) 0.8011071	(*) 0.02063511	(-) 0.7249301	(-) 0.3924872
Australia	France	(-) 0.1447236	(-) 0.7040932	(*) 0.03971054	(-) 0.6347973	(-) 0.3423057
Australia	Korea	(*) 0.002323973	(-) 0.2082155	(*) 1.507028e-08	(-) 0.1529733	(-) 0.5754747
Australia	Turkey	(*) 0.00123133	(-) 0.9202882	(*) 0.002094104	(-) 0.1038833	(-) 0.3810453
Australia	Nigeria	(-) 0.4854854	(-) 0.8742287	(*) 0.04621079	(-) 0.8991949	(-) 0.7040932
Australia	Vt.icj	(-) 0.240239	(-) 0.3585777	(*) 0.03400336	(-) 0.6574948	(-) 0.9243637
Australia	Netherlands	(-) 0.05694258	(-) 0.2107239	(*) 0.02320806	(-) 0.2281173	(-) 0.8894499
Australia	Taiwan	(-) 0.4559448	(-) 0.2340792	(*) 0.008314083	(-) 0.0742022	(-) 0.180862
Australia	Mexico	(-) 0.240239	(-) 0.3585777	(*) 0.03400336	(-) 0.6574948	(-) 0.9243637
Australia	Kenya	(-) 0.3145845	(-) 0.4349975	(*) 0.009281294	(-) 0.8234028	(-) 0.3526988
Unknown	France	(-) 0.09460878	(-) 0.937715	(*) 0.02210578	(-) 0.5095393	(-) 0.4848614
Unknown	Korea	(*) 0.002103348	(*) 0.04480163	(*) 6.403363e-08	(-) 0.2179516	(-) 0.6647749
Unknown	Turkey	(*) 0.003089345	(-) 0.9269902	(*) 0.001379634	(-) 0.08772322	(-) 0.5221758
Unknown	Nigeria	(-) 0.3311281	(-) 0.8762697	(*) 0.02210578	(-) 0.8766453	(-) 0.6980355
Unknown	Vt.icj	(-) 0.102683	(-) 0.3498838	(*) 0.02210578	(-) 0.5346698	(-) 0.6140817
Unknown	Netherlands	(-) 0.07552491	(-) 0.08464388	(*) 0.02564992	(-) 0.4568555	(-) 0.8637114
Unknown	Taiwan	(-) 0.3349214	(-) 0.1684648	(*) 0.00698333	(-) 0.1312419	(-) 0.1433337
Unknown	Mexico	(-) 0.102683	(-) 0.3498838	(*) 0.02210578	(-) 0.5346698	(-) 0.6140817
Unknown	Kenya	(-) 0.1089174	(-) 0.4352778	(*) 0.01191177	(-) 1	(-) 0.3849943
Korea	Nigeria	(-) 0.4373799	(-) 0.2604326	(*) 0.004367841	(-) 0.7559116	(-) 0.5604222
Korea	Iraq	(-) 0.05219	(-) 0.8156523	(*) 0.002047834	(-) 0.3515726	(-) 0.5090769
Turkey	Iraq	(*) 0.00952381	(-) 0.9142857	(*) 0.00952381	(-) 0.1714286	(-) 0.5211659

S3 Comparison of impacts between genotypes

Table S4: The p-values of Mann-Witney-Wilcoxon test for impacts comparison between SNPs. Statistically significant (< 0.05) results are marked with '*'.

Genotype	Genotype	dl	rain	tmax	tmin
snp1AA	snp1AR	(*) 0.0001504167	(-) 0.06990028	(-) 0.1744741	(*) 0.0009554388
snp1AA	snp1RR	(*) 0.0001096401	(-) 0.8202892	(-) 0.8603285	(*) 5.792627e-05
snp1AA	snp2AR	(*) 0.01044522	(*) 0.02515964	(-) 0.06207214	(-) 0.05900161
snp1AA	snp2RR	(*) 0.0001225495	(-) 0.7738752	(-) 0.8186583	(*) 6.506167e-05
snp1AA	snp3AR	(*) 0.002868818	(*) 0.01480745	(*) 0.02805604	(*) 0.02646539
snp1AA	snp3RR	(*) 0.0001541824	(-) 0.7898487	(-) 0.8250423	(*) 8.036174e-05
snp1AA	snp4AR	(*) 0.0008595749	(*) 0.01129763	(*) 0.01932932	(*) 0.01083617
snp1AA	snp4RR	(*) 2.804443e-05	(-) 0.9490947	(-) 0.811996	(*) 1.006348e-05
snp1AA	snp5AA	(*) 0.0003893638	(-) 0.4631712	(-) 0.4177255	(*) 0.001795462
snp1AA	snp5AR	(*) 1.13664e-06	(-) 0.7491823	(-) 0.4335764	(*) 2.769769e-06
snp1AA	snp5RR	(*) 0.004496373	(-) 0.598554	(-) 0.5931661	(*) 0.001409833
snp1AA	snp6AR	(*) 0.009825905	(-) 0.7477205	(-) 0.4164039	(*) 0.03696438
snp1AA	snp6RR	(*) 0.0003341369	(-) 0.6444364	(-) 0.6593132	(*) 0.0001656074
snp1AA	snp7AR	(*) 0.02917048	(*) 0.003304623	(*) 0.00190736	(-) 0.1227477
snp1AA	snp7RR	(*) 2.130383e-06	(-) 0.7441502	(-) 0.488469	(*) 6.397099e-07
snp1AA	snp8AR	(*) 0.0008595749	(*) 0.01129763	(*) 0.01932932	(*) 0.01083617
snp1AA	snp8RR	(*) 3.935484e-06	(-) 0.7950093	(-) 0.5560771	(*) 1.183195e-06
snp1AA	snp9AR	(*) 0.0008595749	(*) 0.01129763	(*) 0.01932932	(*) 0.01083617
snp1AA	snp9RR	(*) 3.935484e-06	(-) 0.7950093	(-) 0.5560771	(*) 1.183195e-06
snp1AA	snp10AA	(*) 0.01053383	(-) 0.4884207	(-) 0.793728	(*) 0.03347734
snp1AA	snp10RR	(*) 0.0001537751	(-) 0.7163914	(-) 0.755606	(*) 7.593172e-05
snp1AR	snp1RR	(*) 0.02690124	(*) 0.01205957	(-) 0.05621615	(-) 0.5243167

snp1AR	snp2AA	(*) 3.274988e-06	(*) 0.01841108	(-) 0.06551418	(*) 0.0001083831
snp1AR	snp2RR	(*) 0.02455819	(*) 0.01468115	(-) 0.06359526	(-) 0.5436952
snp1AR	snp3AA	(*) 2.673865e-06	(*) 0.01628708	(*) 0.03686165	(*) 8.543249e-05
snp1AR	snp3RR	(*) 0.0225323	(*) 0.01412014	(-) 0.06489157	(-) 0.4999943
snp1AR	snp4AA	(*) 9.846065e-07	(*) 0.01734957	(*) 0.03692571	(*) 2.899085e-05
snp1AR	snp4RR	(-) 0.0529625	(*) 0.004235636	(*) 0.01887647	(-) 0.7935702
snp1AR	snp5AR	(-) 0.3985956	(*) 0.002331684	(*) 0.006218512	(-) 0.5721406
snp1AR	snp5RR	(*) 0.003752593	(*) 0.04560621	(-) 0.1556657	(-) 0.1627629
snp1AR	snp6AR	(-) 0.1209153	(*) 0.01174072	(*) 0.01645279	(-) 0.07678043
snp1AR	snp6RR	(*) 0.01541123	(*) 0.02735928	(-) 0.1109467	(-) 0.4065464
snp1AR	snp7AA	(*) 2.502698e-07	(-) 0.05854538	(-) 0.1214443	(*) 8.302554e-06
snp1AR	snp7RR	(-) 0.1207957	(*) 0.001268333	(*) 0.003768524	(-) 0.8525374
snp1AR	snp8AA	(*) 9.846065e-07	(*) 0.01734957	(*) 0.03692571	(*) 2.899085e-05
snp1AR	snp8RR	(-) 0.09982436	(*) 0.001749709	(*) 0.005467564	(-) 0.9328896
snp1AR	snp9AA	(*) 9.846065e-07	(*) 0.01734957	(*) 0.03692571	(*) 2.899085e-05
snp1AR	snp9RR	(-) 0.09982436	(*) 0.001749709	(*) 0.005467564	(-) 0.9328896
snp1AR	snp10AR	(*) 0.03229088	(*) 0.02220957	(-) 0.05204617	(*) 0.02574509
snp1AR	snp10RR	(*) 0.02428347	(*) 0.02020596	(-) 0.08146667	(-) 0.528851
snp1RR	snp2AA	(*) 2.243085e-07	(-) 0.2468899	(-) 0.31124	(*) 4.912321e-06
snp1RR	snp2AR	(-) 0.5252533	(*) 0.002280376	(*) 0.00554182	(-) 0.1254122
snp1RR	snp3AA	(*) 1.683314e-07	(-) 0.2404236	(-) 0.182993	(*) 2.663446e-06
snp1RR	snp3AR	(-) 0.1453192	(*) 0.001070787	(*) 0.001793545	(-) 0.410335
snp1RR	snp4AA	(*) 2.085452e-08	(-) 0.2826827	(-) 0.1884016	(*) 3.486802e-07
snp1RR	snp4AR	(*) 0.03949528	(*) 0.0008398624	(*) 0.001078117	(-) 0.7661619
snp1RR	snp5AR	(*) 0.01451192	(-) 0.339779	(-) 0.06682581	(*) 0.03622976
snp1RR	snp5RR	(*) 0.01782452	(-) 0.4263589	(-) 0.2749939	(*) 0.04379295
snp1RR	snp7AA	(*) 1.663832e-09	(-) 0.6063716	(-) 0.5316839	(*) 3.262075e-08
snp1RR	snp7AR	(-) 0.6254041	(*) 7.16719e-05	(*) 5.295881e-06	(-) 0.1048947
snp1RR	snp7RR	(*) 0.0323763	(-) 0.1288041	(*) 0.009585685	(*) 0.02031283
snp1RR	snp8AA	(*) 2.085452e-08	(-) 0.2826827	(-) 0.1884016	(*) 3.486802e-07
snp1RR	snp8AR	(*) 0.03949528	(*) 0.0008398624	(*) 0.001078117	(-) 0.7661619
snp1RR	snp8RR	(-) 0.06596817	(-) 0.1806271	(*) 0.0229673	(*) 0.04168592
snp1RR	snp9AA	(*) 2.085452e-08	(-) 0.2826827	(-) 0.1884016	(*) 3.486802e-07
snp1RR	snp9AR	(*) 0.03949528	(*) 0.0008398624	(*) 0.001078117	(-) 0.7661619
snp1RR	snp9RR	(-) 0.06596817	(-) 0.1806271	(*) 0.0229673	(*) 0.04168592
snp1RR	snp10AA	(*) 0.04286874	(-) 0.3891316	(-) 0.7714987	(-) 0.2495724
snp1RR	snp10AR	(-) 0.176658	(-) 0.5674042	(-) 0.396307	(*) 0.01002459
snp2AA	snp2AR	(*) 0.0007192304	(*) 0.008938525	(*) 0.02138383	(*) 0.00985642
snp2AA	snp2RR	(*) 2.563201e-07	(-) 0.226584	(-) 0.2921736	(*) 6.041104e-06
snp2AA	snp3AR	(*) 0.000211112	(*) 0.005008861	(*) 0.008847459	(*) 0.004067892
snp2AA	snp3RR	(*) 3.443413e-07	(-) 0.2349629	(-) 0.2972245	(*) 7.23082e-06
snp2AA	snp4AR	(*) 5.937456e-05	(*) 0.003787033	(*) 0.005718271	(*) 0.001513193
snp2AA	snp4RR	(*) 4.383933e-08	(-) 0.3489264	(-) 0.5091993	(*) 8.009502e-07
snp2AA	snp5AA	(*) 2.038944e-06	(-) 0.1311628	(-) 0.1392579	(*) 0.0002278822
snp2AA	snp5AR	(*) 9.191107e-10	(-) 0.4545779	(-) 0.926327	(*) 1.99927e-07
snp2AA	snp5RR	(*) 2.702676e-05	(-) 0.1820763	(-) 0.1950357	(*) 0.0001224219
snp2AA	snp6AR	(*) 0.0002504458	(-) 0.5424439	(-) 0.9795282	(*) 0.004094286
snp2AA	snp6RR	(*) 9.235691e-07	(-) 0.1836304	(-) 0.2217415	(*) 1.551845e-05
snp2AA	snp7AR	(*) 0.003615288	(*) 0.001508256	(*) 0.0005439933	(*) 0.03199387
snp2AA	snp7RR	(*) 1.898473e-09	(-) 0.4438861	(-) 0.7688572	(*) 4.520064e-08
snp2AA	snp8AR	(*) 5.937456e-05	(*) 0.003787033	(*) 0.005718271	(*) 0.001513193
snp2AA	snp8RR	(*) 3.933176e-09	(-) 0.4173961	(-) 0.7032051	(*) 8.593345e-08
snp2AA	snp9AR	(*) 5.937456e-05	(*) 0.003787033	(*) 0.005718271	(*) 0.001513193
snp2AA	snp9RR	(*) 3.933176e-09	(-) 0.4173961	(-) 0.7032051	(*) 8.593345e-08
snp2AA	snp10AA	(*) 0.002790185	(-) 0.348656	(-) 0.6390248	(*) 0.01364108
snp2AA	snp10AR	(*) 0.004104477	(-) 0.4955287	(-) 0.7334016	(*) 0.02643918
snp2AA	snp10RR	(*) 3.553952e-07	(-) 0.2087526	(-) 0.2667062	(*) 7.040686e-06
snp2AR	snp2RR	(-) 0.5011792	(*) 0.002823066	(*) 0.006601284	(-) 0.1226149
snp2AR	snp3AA	(*) 0.0006299042	(*) 0.00713604	(*) 0.01068154	(*) 0.00922677
snp2AR	snp3RR	(-) 0.4816605	(*) 0.00272424	(*) 0.006810512	(-) 0.1390333
snp2AR	snp4AA	(*) 0.0003414319	(*) 0.006879302	(*) 0.009669997	(*) 0.004498946
snp2AR	snp4RR	(-) 0.7189092	(*) 0.0007063917	(*) 0.001302509	(-) 0.0536936
snp2AR	snp5AA	(-) 0.7082706	(*) 0.0351139	(-) 0.08396118	(-) 0.3233467
snp2AR	snp5AR	(-) 0.6015358	(*) 0.0003489972	(*) 0.0005008255	(*) 0.0119506
snp2AR	snp5RR	(-) 0.1847873	(*) 0.01217907	(*) 0.02392914	(-) 0.4829447
snp2AR	snp6AR	(-) 0.6857384	(*) 0.002263827	(*) 0.002280776	(-) 0.9274772
snp2AR	snp6RR	(-) 0.3947311	(*) 0.005962829	(*) 0.01419828	(-) 0.1914046
snp2AR	snp7AA	(*) 0.0001195049	(*) 0.0248185	(*) 0.04057599	(*) 0.001689463
snp2AR	snp7RR	(-) 0.9575187	(*) 0.0001709246	(*) 0.0001651324	(*) 0.01466022
snp2AR	snp8AA	(*) 0.0003414319	(*) 0.006879302	(*) 0.009669997	(*) 0.004498946
snp2AR	snp8RR	(-) 0.9646964	(*) 0.0002502894	(*) 0.0002614414	(*) 0.01990896
snp2AR	snp9AA	(*) 0.0003414319	(*) 0.006879302	(*) 0.009669997	(*) 0.004498946
snp2AR	snp9RR	(-) 0.9646964	(*) 0.0002502894	(*) 0.0002614414	(*) 0.01990896
snp2AR	snp10AR	(-) 0.3016445	(*) 0.006121981	(*) 0.01030898	(-) 0.5301335
snp2AR	snp10RR	(-) 0.4933956	(*) 0.004062923	(*) 0.00945629	(-) 0.1317497
snp2RR	snp3AA	(*) 1.9219e-07	(-) 0.2199535	(-) 0.1698729	(*) 3.287577e-06
snp2RR	snp3AR	(-) 0.1352981	(*) 0.0013274	(*) 0.00218191	(-) 0.4021379
snp2RR	snp4AA	(*) 2.413167e-08	(-) 0.2586848	(-) 0.175059	(*) 4.442995e-07
snp2RR	snp4AR	(*) 0.03612958	(*) 0.001038531	(*) 0.001326843	(-) 0.751256
snp2RR	snp5AR	(*) 0.01292698	(-) 0.2819626	(-) 0.05403329	(*) 0.04221896
snp2RR	snp5RR	(*) 0.02011112	(-) 0.5273382	(-) 0.3392399	(*) 0.03653082
snp2RR	snp7AA	(*) 1.962159e-09	(-) 0.5684286	(-) 0.5054483	(*) 4.377938e-08
snp2RR	snp7AR	(-) 0.6001662	(*) 9.067437e-05	(*) 7.200862e-06	(-) 0.1047001
snp2RR	snp7RR	(*) 0.02639507	(-) 0.08349181	(*) 0.005580846	(*) 0.02606307
snp2RR	snp8AA	(*) 2.413167e-08	(-) 0.2586848	(-) 0.175059	(*) 4.442995e-07
snp2RR	snp8AR	(*) 0.03612958	(*) 0.001038531	(*) 0.001326843	(-) 0.751256
snp2RR	snp8RR	(-) 0.05529844	(-) 0.1215835	(*) 0.01415696	(-) 0.05199125
snp2RR	snp9AA	(*) 2.413167e-08	(-) 0.2586848	(-) 0.175059	(*) 4.442995e-07
snp2RR	snp9AR	(*) 0.03612958	(*) 0.001038531	(*) 0.001326843	(-) 0.751256
snp2RR	snp9RR	(-) 0.05529844	(-) 0.1215835	(*) 0.01415696	(-) 0.05199125
snp2RR	snp10AA	(*) 0.04271928	(-) 0.4010587	(-) 0.7835794	(-) 0.2559624
snp2RR	snp10AR	(-) 0.1884185	(-) 0.5203282	(-) 0.3632962	(*) 0.01016481
snp3AA	snp3AR	(*) 0.0001695598	(*) 0.004014737	(*) 0.004070715	(*) 0.003726102
snp3AA	snp3RR	(*) 2.61939e-07	(-) 0.2280946	(-) 0.173621	(*) 3.984426e-06
snp3AA	snp4AR	(*) 4.453047e-05	(*) 0.003058863	(*) 0.002608101	(*) 0.001331773
snp3AA	snp4RR	(*) 3.123292e-08	(-) 0.3473627	(-) 0.332813	(*) 3.896749e-07
snp3AA	snp5AA	(*) 1.629724e-06	(-) 0.1255653	(-) 0.07609517	(*) 0.0001687035
snp3AA	snp5AR	(*) 6.737589e-10	(-) 0.4748219	(-) 0.7140958	(*) 1.042576e-07
snp3AA	snp5RR	(*) 2.492092e-05	(-) 0.1711064	(-) 0.106921	(*) 8.243122e-05
snp3AA	snp6AR	(*) 0.0002676423	(-) 0.5669218	(-) 0.7950206	(*) 0.003859272
snp3AA	snp6RR	(*) 7.259093e-07	(-) 0.1747805	(-) 0.1225547	(*) 8.950585e-06
snp3AA	snp7AR	(*) 0.003031666	(*) 0.001111637	(*) 0.0001999731	(*) 0.03017171
snp3AA	snp7RR	(*) 1.21503e-09	(-) 0.451611	(-) 0.551332	(*) 1.833663e-08
snp3AA	snp8AR	(*) 4.453047e-05	(*) 0.003058863	(*) 0.002608101	(*) 0.001331773
snp3AA	snp8RR	(*) 2.579546e-09	(-) 0.4229938	(-) 0.4934043	(*) 3.622204e-08
snp3AA	snp9AR	(*) 4.453047e-05	(*) 0.003058863	(*) 0.002608101	(*) 0.001331773
snp3AA	snp9RR	(*) 2.579546e-09	(-) 0.4229938	(-) 0.4934043	(*) 3.622204e-08
snp3AA	snp10AA	(*) 0.002762831	(-) 0.3429722	(-) 0.5804308	(*) 0.0132654
snp3AA	snp10AR	(*) 0.004682867	(-) 0.5239362	(-) 0.5717338	(*) 0.02658406
snp3AA	snp10RR	(*) 2.681455e-07	(-) 0.2001566	(-) 0.1527826	(*) 3.849272e-06

snp3AR	snp3RR	(-) 0.1290644	(*) 0.001282614	(*) 0.002277616	(-) 0.4368884
snp3AR	snp4AA	(*) 9.019058e-05	(*) 0.003880707	(*) 0.00362833	(*) 0.001754852
snp3AR	snp4RR	(-) 0.227648	(*) 0.0003296785	(*) 0.000385471	(-) 0.23382
snp3AR	snp5AA	(-) 0.2643661	(*) 0.01868815	(*) 0.03728834	(-) 0.6776017
snp3AR	snp5AR	(-) 0.7446938	(*) 0.0001572341	(*) 0.0001338118	(-) 0.06703055
snp3AR	snp5RR	(*) 0.04098406	(*) 0.00591445	(*) 0.009140916	(-) 0.9436624
snp3AR	snp6AR	(-) 0.3341202	(*) 0.001158889	(*) 0.0006660839	(-) 0.5795248
snp3AR	snp6RR	(-) 0.0996931	(*) 0.002840529	(*) 0.005094237	(-) 0.5356139
snp3AR	snp7AA	(*) 3.131767e-05	(*) 0.01481319	(*) 0.01826723	(*) 0.0006533279
snp3AR	snp7RR	(-) 0.3965076	(*) 7.937832e-05	(*) 4.145744e-05	(-) 0.0980571
snp3AR	snp8AA	(*) 9.019058e-05	(*) 0.003880707	(*) 0.00362833	(*) 0.001754852
snp3AR	snp8RR	(-) 0.3499103	(*) 0.0001164041	(*) 6.812326e-05	(-) 0.1208828
snp3AR	snp9AA	(*) 9.019058e-05	(*) 0.003880707	(*) 0.00362833	(*) 0.001754852
snp3AR	snp9RR	(-) 0.3499103	(*) 0.0001164041	(*) 6.812326e-05	(-) 0.1208828
snp3AR	snp10AR	(-) 0.1261	(*) 0.003176329	(*) 0.003601429	(-) 0.29759
snp3AR	snp10RR	(-) 0.1341025	(*) 0.001932073	(*) 0.003268268	(-) 0.4209897
snp3RR	snp4AA	(*) 3.387224e-08	(-) 0.2674181	(-) 0.1784477	(*) 5.490916e-07
snp3RR	snp4AR	(*) 0.0342757	(*) 0.001002013	(*) 0.001386279	(-) 0.7972786
snp3RR	snp5AR	(*) 0.01015057	(-) 0.2989113	(-) 0.05341253	(*) 0.03177358
snp3RR	snp5RR	(*) 0.02792548	(-) 0.4905514	(-) 0.3422081	(-) 0.0540176
snp3RR	snp7AA	(*) 2.812561e-09	(-) 0.5813505	(-) 0.5111351	(*) 5.511319e-08
snp3RR	snp7AR	(-) 0.5837084	(*) 8.69955e-05	(*) 7.821626e-06	(-) 0.1181188
snp3RR	snp7RR	(*) 0.01756115	(-) 0.09508558	(*) 0.005398982	(*) 0.01518237
snp3RR	snp8AA	(*) 3.387224e-08	(-) 0.2674181	(-) 0.1784477	(*) 5.490916e-07
snp3RR	snp8AR	(*) 0.0342757	(*) 0.001002013	(*) 0.001386279	(-) 0.7972786
snp3RR	snp8RR	(*) 0.03847539	(-) 0.1371879	(*) 0.01373045	(*) 0.03199342
snp3RR	snp9AA	(*) 3.387224e-08	(-) 0.2674181	(-) 0.1784477	(*) 5.490916e-07
snp3RR	snp9AR	(*) 0.0342757	(*) 0.001002013	(*) 0.001386279	(-) 0.7972786
snp3RR	snp9RR	(*) 0.03847539	(-) 0.1371879	(*) 0.01373045	(*) 0.03199342
snp3RR	snp10AA	(*) 0.04209391	(-) 0.4013399	(-) 0.7847878	(-) 0.2469899
snp3RR	snp10AR	(-) 0.2056731	(-) 0.5371865	(-) 0.3666042	(*) 0.01207843
snp4AA	snp4AR	(*) 2.275552e-05	(*) 0.002932825	(*) 0.00230973	(*) 0.0005852463
snp4AA	snp4RR	(*) 3.201906e-09	(-) 0.4091044	(-) 0.349499	(*) 4.093161e-08
snp4AA	snp5AA	(*) 3.642625e-07	(-) 0.1432768	(-) 0.07521242	(*) 4.607268e-05
snp4AA	snp5AR	(*) 6.511542e-11	(-) 0.5723033	(-) 0.7616128	(*) 1.29558e-08
snp4AA	snp5RR	(*) 5.543917e-06	(-) 0.1937079	(-) 0.1064993	(*) 1.672699e-05
snp4AA	snp6AR	(*) 0.0001110351	(-) 0.631338	(-) 0.8217843	(*) 0.00157835
snp4AA	snp6RR	(*) 1.060207e-07	(-) 0.2033517	(-) 0.1238564	(*) 1.364824e-06
snp4AA	snp7AR	(*) 0.00194995	(*) 0.0009952684	(*) 0.0001528099	(*) 0.01743841
snp4AA	snp7RR	(*) 8.553267e-11	(-) 0.5337876	(-) 0.5890763	(*) 1.341386e-09
snp4AA	snp8AR	(*) 2.275552e-05	(*) 0.002932825	(*) 0.00230973	(*) 0.0005852463
snp4AA	snp8RR	(*) 1.97669e-10	(-) 0.5001372	(-) 0.5259617	(*) 2.87106e-09
snp4AA	snp9AR	(*) 2.275552e-05	(*) 0.002932825	(*) 0.00230973	(*) 0.0005852463
snp4AA	snp9RR	(*) 1.97669e-10	(-) 0.5001372	(-) 0.5259617	(*) 2.87106e-09
snp4AA	snp10AA	(*) 0.002469421	(-) 0.3300884	(-) 0.5866854	(*) 0.0108189
snp4AA	snp10AR	(*) 0.002540858	(-) 0.5877067	(-) 0.5846072	(*) 0.0140988
snp4AA	snp10RR	(*) 3.50334e-08	(-) 0.2330724	(-) 0.1567168	(*) 5.280122e-07
snp4AR	snp4RR	(-) 0.06808646	(*) 0.0002645945	(*) 0.0002309554	(-) 0.5151766
snp4AR	snp5AA	(-) 0.1001534	(*) 0.01409867	(*) 0.0242643	(-) 0.9843297
snp4AR	snp5AR	(-) 0.3465703	(*) 0.0001287469	(*) 8.296601e-05	(-) 0.1818494
snp4AR	snp5RR	(*) 0.00964668	(*) 0.004499501	(*) 0.005681178	(-) 0.6720365
snp4AR	snp6AR	(-) 0.1648771	(*) 0.0008263442	(*) 0.0003946792	(-) 0.3333426
snp4AR	snp6RR	(*) 0.02552056	(*) 0.002194606	(*) 0.003134083	(-) 0.9150812
snp4AR	snp7AA	(*) 7.915971e-06	(*) 0.01121073	(*) 0.01229171	(*) 0.00020909
snp4AR	snp7RR	(-) 0.1340833	(*) 6.514297e-05	(*) 2.489607e-05	(-) 0.2763066
snp4AR	snp8AA	(*) 2.275552e-05	(*) 0.002932825	(*) 0.00230973	(*) 0.0005852463
snp4AR	snp8RR	(-) 0.1145106	(*) 9.521614e-05	(*) 4.077252e-05	(-) 0.3217239
snp4AR	snp9AA	(*) 2.275552e-05	(*) 0.002932825	(*) 0.00230973	(*) 0.0005852463
snp4AR	snp9RR	(-) 0.1145106	(*) 9.521614e-05	(*) 4.077252e-05	(-) 0.3217239
snp4AR	snp10AR	(-) 0.05529998	(*) 0.002394311	(*) 0.002201051	(-) 0.156223
snp4AR	snp10RR	(*) 0.03615972	(*) 0.001489708	(*) 0.002016284	(-) 0.7761583
snp4RR	snp5AA	(-) 0.8897525	(-) 0.1147751	(*) 0.04947677	(-) 0.2752679
snp4RR	snp5RR	(*) 0.001689267	(-) 0.1254586	(*) 0.03048613	(*) 0.00288452
snp4RR	snp6AR	(-) 0.5037843	(-) 0.7598676	(-) 0.3497121	(*) 0.01577441
snp4RR	snp6RR	(-) 0.1239341	(-) 0.1287601	(*) 0.0436167	(-) 0.1060577
snp4RR	snp7AA	(*) 2.181093e-10	(-) 0.79487	(-) 0.8081566	(*) 3.314922e-09
snp4RR	snp7AR	(-) 0.8076272	(*) 1.949356e-05	(*) 5.917101e-07	(*) 0.04774811
snp4RR	snp8AA	(*) 3.201906e-09	(-) 0.4091044	(-) 0.349499	(*) 4.093161e-08
snp4RR	snp8AR	(-) 0.06808646	(*) 0.0002645945	(*) 0.0002309554	(-) 0.5151766
snp4RR	snp9AA	(*) 3.201906e-09	(-) 0.4091044	(-) 0.349499	(*) 4.093161e-08
snp4RR	snp9AR	(-) 0.06808646	(*) 0.0002645945	(*) 0.0002309554	(-) 0.5151766
snp4RR	snp10AA	(*) 0.04783594	(-) 0.3414158	(-) 0.6806946	(-) 0.2870937
snp4RR	snp10AR	(-) 0.09218518	(-) 0.813024	(-) 0.7113788	(*) 0.002359533
snp5AA	snp5AR	(-) 0.2061911	(-) 0.09263423	(*) 0.01521661	(-) 0.05600474
snp5AA	snp6AR	(-) 0.6260447	(-) 0.1927043	(*) 0.04077273	(-) 0.2408304
snp5AA	snp7AA	(*) 5.875987e-08	(-) 0.3489955	(-) 0.2498162	(*) 8.340793e-06
snp5AA	snp7AR	(-) 0.7802919	(*) 0.002485422	(*) 0.0008385229	(-) 0.2563429
snp5AA	snp7RR	(-) 0.5830162	(*) 0.04251301	(*) 0.004992653	(-) 0.06731003
snp5AA	snp8AA	(*) 3.642625e-07	(-) 0.1432768	(-) 0.07521242	(*) 4.607268e-05
snp5AA	snp8AR	(-) 0.1001534	(*) 0.01409867	(*) 0.0242643	(-) 0.9843297
snp5AA	snp8RR	(-) 0.7011318	(-) 0.05572145	(*) 0.008768294	(-) 0.09596277
snp5AA	snp9AA	(*) 3.642625e-07	(-) 0.1432768	(-) 0.07521242	(*) 4.607268e-05
snp5AA	snp9AR	(-) 0.1001534	(*) 0.01409867	(*) 0.0242643	(-) 0.9843297
snp5AA	snp9RR	(-) 0.7011318	(-) 0.05572145	(*) 0.008768294	(-) 0.09596277
snp5AR	snp5RR	(*) 0.0001263379	(-) 0.1519589	(*) 0.01471302	(*) 0.001080492
snp5AR	snp6AR	(-) 0.08432686	(-) 0.9366879	(-) 0.946102	(*) 0.002980046
snp5AR	snp6RR	(*) 0.00449014	(-) 0.1674284	(*) 0.02039362	(*) 0.01604307
snp5AR	snp7AA	(*) 5.578792e-12	(-) 0.9974139	(-) 0.7472624	(*) 1.409048e-09
snp5AR	snp7AR	(-) 0.6124175	(*) 1.239417e-05	(*) 3.548399e-07	(*) 0.01168341
snp5AR	snp8AA	(*) 6.511542e-11	(-) 0.5723033	(-) 0.7616128	(*) 1.29558e-08
snp5AR	snp8AR	(-) 0.3465703	(*) 0.0001287469	(*) 8.296601e-05	(-) 0.1818494
snp5AR	snp9AA	(*) 6.511542e-11	(-) 0.5723033	(-) 0.7616128	(*) 1.29558e-08
snp5AR	snp9AR	(-) 0.3465703	(*) 0.0001287469	(*) 8.296601e-05	(-) 0.1818494
snp5AR	snp10AR	(*) 0.009962862	(-) 0.9509956	(-) 0.7257356	(*) 0.0004443153
snp5AR	snp10RR	(*) 0.01163547	(-) 0.217107	(*) 0.03860707	(*) 0.03950334
snp5RR	snp7AA	(*) 7.11411e-07	(-) 0.4380727	(-) 0.3488644	(*) 2.357071e-06
snp5RR	snp7AR	(-) 0.28534	(*) 0.0005504756	(*) 6.963217e-05	(-) 0.3950443
snp5RR	snp7RR	(*) 1.126197e-05	(*) 0.03063231	(*) 0.0005184344	(*) 2.963799e-05
snp5RR	snp8AA	(*) 5.543917e-06	(-) 0.1937079	(-) 0.1064993	(*) 1.672699e-05
snp5RR	snp8AR	(*) 0.00964668	(*) 0.004499501	(*) 0.005681178	(-) 0.6720365
snp5RR	snp8RR	(*) 3.987116e-05	(*) 0.04593388	(*) 0.001447505	(*) 9.097866e-05
snp5RR	snp9AA	(*) 5.543917e-06	(-) 0.1937079	(-) 0.1064993	(*) 1.672699e-05
snp5RR	snp9AR	(*) 0.00964668	(*) 0.004499501	(*) 0.005681178	(-) 0.6720365
snp5RR	snp9RR	(*) 3.987116e-05	(*) 0.04593388	(*) 0.001447505	(*) 9.097866e-05
snp5RR	snp10AA	(*) 0.03242748	(-) 0.4837333	(-) 0.8847847	(-) 0.1783953
snp5RR	snp10RR	(*) 0.02565332	(-) 0.6786655	(-) 0.4830009	(*) 0.04503924
snp6AR	snp7AA	(*) 2.858249e-05	(-) 0.967973	(-) 0.7390161	(*) 0.0004221321
snp6AR	snp7AR	(-) 0.8477975	(*) 0.0001336692	(*) 6.020137e-06	(-) 0.8724476
snp6AR	snp7RR	(-) 0.2211373	(-) 0.971635	(-) 0.7244504	(*) 0.002326986
snp6AR	snp8AA	(*) 0.0001110351	(-) 0.631338	(-) 0.8217843	(*) 0.00157835

snp6AR	snp8AR	(-) 0.1648771	(*) 0.0008263442	(*) 0.0003946792	(-) 0.3333426
snp6AR	snp8RR	(-) 0.2731952	(-) 0.9621532	(-) 0.6244394	(*) 0.00367307
snp6AR	snp9AA	(*) 0.0001110351	(-) 0.631338	(-) 0.8217843	(*) 0.00157835
snp6AR	snp9AR	(-) 0.1648771	(*) 0.0008263442	(*) 0.0003946792	(-) 0.3333426
snp6AR	snp9RR	(-) 0.2731952	(-) 0.9621532	(-) 0.6244394	(*) 0.00367307
snp6RR	snp7AA	(*) 9.717903e-09	(-) 0.4701799	(-) 0.3940291	(*) 1.480852e-07
snp6RR	snp7AR	(-) 0.4998739	(*) 0.0002156567	(*) 2.584471e-05	(-) 0.1596932
snp6RR	snp7RR	(*) 0.004170141	(*) 0.02574029	(*) 0.0005309084	(*) 0.003786539
snp6RR	snp8AA	(*) 1.060207e-07	(-) 0.2033517	(-) 0.1238564	(*) 1.364824e-06
snp6RR	snp8AR	(*) 0.02552056	(*) 0.002194606	(*) 0.003134083	(-) 0.9150812
snp6RR	snp8RR	(*) 0.01049002	(*) 0.04081785	(*) 0.001642358	(*) 0.009025005
snp6RR	snp9AA	(*) 1.060207e-07	(-) 0.2033517	(-) 0.1238564	(*) 1.364824e-06
snp6RR	snp9AR	(*) 0.02552056	(*) 0.002194606	(*) 0.003134083	(-) 0.9150812
snp6RR	snp9RR	(*) 0.01049002	(*) 0.04081785	(*) 0.001642358	(*) 0.009025005
snp6RR	snp10AA	(*) 0.04041485	(-) 0.4421013	(-) 0.8404622	(-) 0.2321283
snp6RR	snp10AR	(-) 0.2836574	(-) 0.4013524	(-) 0.2459252	(*) 0.02087591
snp7AA	snp7AR	(*) 0.0008439676	(*) 0.003874413	(*) 0.001344666	(*) 0.007659752
snp7AA	snp7RR	(*) 4.265222e-12	(-) 0.9684704	(-) 0.8755714	(*) 8.205067e-11
snp7AA	snp8AR	(*) 7.915971e-06	(*) 0.01121073	(*) 0.01229171	(*) 0.00020909
snp7AA	snp8RR	(*) 1.054448e-11	(-) 0.9231603	(-) 0.948811	(*) 1.854744e-10
snp7AA	snp9AR	(*) 7.915971e-06	(*) 0.01121073	(*) 0.01229171	(*) 0.00020909
snp7AA	snp9RR	(*) 1.054448e-11	(-) 0.9231603	(-) 0.948811	(*) 1.854744e-10
snp7AA	snp10AA	(*) 0.002293246	(-) 0.4586528	(-) 0.7390318	(*) 0.009429634
snp7AA	snp10AR	(*) 0.0008887887	(-) 0.9444774	(-) 0.9964161	(*) 0.004773023
snp7AA	snp10RR	(*) 2.924768e-09	(-) 0.5255115	(-) 0.4641418	(*) 5.325541e-08
snp7AR	snp7RR	(-) 0.9103525	(*) 4.008357e-06	(*) 2.467426e-08	(*) 0.01404489
snp7AR	snp8AA	(*) 0.00194995	(*) 0.0009952684	(*) 0.0001528099	(*) 0.01743841
snp7AR	snp8RR	(-) 0.9802089	(*) 6.225417e-06	(*) 4.859968e-08	(*) 0.01864772
snp7AR	snp9AA	(*) 0.00194995	(*) 0.0009952684	(*) 0.0001528099	(*) 0.01743841
snp7AR	snp9RR	(-) 0.9802089	(*) 6.225417e-06	(*) 4.859968e-08	(*) 0.01864772
snp7AR	snp10AR	(-) 0.4468304	(*) 0.0005277179	(*) 7.076133e-05	(-) 0.7555406
snp7AR	snp10RR	(-) 0.5970651	(*) 0.0001375655	(*) 1.385462e-05	(-) 0.1131848
snp7RR	snp8AA	(*) 8.553267e-11	(-) 0.5337876	(-) 0.5890763	(*) 1.341386e-09
snp7RR	snp8AR	(-) 0.1340833	(*) 6.514297e-05	(*) 2.489607e-05	(-) 0.2763066
snp7RR	snp9AA	(*) 8.553267e-11	(-) 0.5337876	(-) 0.5890763	(*) 1.341386e-09
snp7RR	snp9AR	(-) 0.1340833	(*) 6.514297e-05	(*) 2.489607e-05	(-) 0.2763066
snp7RR	snp10AR	(*) 0.02647197	(-) 0.9563376	(-) 0.866487	(*) 0.0002583586
snp7RR	snp10RR	(*) 0.02186932	(*) 0.04821793	(*) 0.002609848	(*) 0.02235186
snp8AA	snp8AR	(*) 2.275552e-05	(*) 0.002932825	(*) 0.00230973	(*) 0.0005852463
snp8AA	snp8RR	(*) 1.97669e-10	(-) 0.5001372	(-) 0.5259617	(*) 2.87106e-09
snp8AA	snp9AR	(*) 2.275552e-05	(*) 0.002932825	(*) 0.00230973	(*) 0.0005852463
snp8AA	snp9RR	(*) 1.97669e-10	(-) 0.5001372	(-) 0.5259617	(*) 2.87106e-09
snp8AA	snp10AA	(*) 0.002469421	(-) 0.3300884	(-) 0.5866854	(*) 0.0108189
snp8AA	snp10AR	(*) 0.002540858	(-) 0.5877067	(-) 0.5846072	(*) 0.0140988
snp8AA	snp10RR	(*) 3.50334e-08	(-) 0.2330724	(-) 0.1567168	(*) 5.280122e-07
snp8AR	snp8RR	(-) 0.1145106	(*) 9.521614e-05	(*) 4.077252e-05	(-) 0.3217239
snp8AR	snp9AA	(*) 2.275552e-05	(*) 0.002932825	(*) 0.00230973	(*) 0.0005852463
snp8AR	snp9RR	(-) 0.1145106	(*) 9.521614e-05	(*) 4.077252e-05	(-) 0.3217239
snp8AR	snp10AR	(-) 0.05529998	(*) 0.002394311	(*) 0.002201051	(-) 0.156223
snp8AR	snp10RR	(*) 0.03615972	(*) 0.001489708	(*) 0.002016284	(-) 0.7761583
snp8RR	snp9AA	(*) 1.97669e-10	(-) 0.5001372	(-) 0.5259617	(*) 2.87106e-09
snp8RR	snp9AR	(-) 0.1145106	(*) 9.521614e-05	(*) 4.077252e-05	(-) 0.3217239
snp8RR	snp10AR	(*) 0.03606379	(-) 0.9850719	(-) 0.9662691	(*) 0.0004329259
snp8RR	snp10RR	(*) 0.04657232	(-) 0.07301441	(*) 0.006999731	(*) 0.04504181
snp9AA	snp9AR	(*) 2.275552e-05	(*) 0.002932825	(*) 0.00230973	(*) 0.0005852463
snp9AA	snp9RR	(*) 1.97669e-10	(-) 0.5001372	(-) 0.5259617	(*) 2.87106e-09
snp9AA	snp10AA	(*) 0.002469421	(-) 0.3300884	(-) 0.5866854	(*) 0.0108189
snp9AA	snp10AR	(*) 0.002540858	(-) 0.5877067	(-) 0.5846072	(*) 0.0140988
snp9AA	snp10RR	(*) 3.50334e-08	(-) 0.2330724	(-) 0.1567168	(*) 5.280122e-07
snp9AR	snp9RR	(-) 0.1145106	(*) 9.521614e-05	(*) 4.077252e-05	(-) 0.3217239
snp9AR	snp10AR	(-) 0.05529998	(*) 0.002394311	(*) 0.002201051	(-) 0.156223
snp9AR	snp10RR	(*) 0.03615972	(*) 0.001489708	(*) 0.002016284	(-) 0.7761583
snp9RR	snp10AR	(*) 0.03606379	(-) 0.9850719	(-) 0.9662691	(*) 0.0004329259
snp9RR	snp10RR	(*) 0.04657232	(-) 0.07301441	(*) 0.006999731	(*) 0.04504181
snp10AA	snp10AR	(*) 0.04470972	(-) 0.3705038	(-) 0.6583527	(-) 0.08287341
snp10AA	snp10RR	(*) 0.043231	(-) 0.4186766	(-) 0.8003062	(-) 0.2504881
snp10AR	snp10RR	(-) 0.2025579	(-) 0.4623893	(-) 0.3145608	(*) 0.01125403

Forecasting

```
delta y= 0.639634* (srad - 9.98812)/(tmin * tmin)
+4.14719* 1/(dl - 0.353241)
+0.00233974* (1/(srad - 9.98812) - 1/(dl - 0.353241)) + 1/(rain - 2.07446))
+4.345e-06*rain
+3e-06*rain /( 1/(dl - 0.353241)  +  tmin )
  -8.86142*snp1AR*f[1]-8.39551*snp1RR*f[1]-6.16543*snp2AA*f[1]-6.48901*snp2RR*f[1]
  -9.25132*snp3AA*f[1]-9.94413*snp3AR*f[1]+6.66347*snp4AR*f[1]-9.22408*snp5AA*f[1]
  -9.98304*snp5RR*f[1]-9.96162*snp6AR*f[1]-9.53273*snp7AA*f[1]+9.47996*snp7AR*f[1]
  +7.34938*snp8AR*f[1]-7.09164*snp8RR*f[1]+6.99908*snp9AA*f[1]+7.36435*snp10RR*f[1]
  +8.42357*snp1AR*f[2]-7.23517*snp2AR*f[2]-9.98429*snp3AR*f[2]+9.85827*snp4AA*f[2]
  -7.64458*snp4RR*f[2]-9.22494*snp5AA*f[2]+9.80398*snp5AR*f[2]+8.28564*snp6AA*f[2]
  +9.04119*snp6AR*f[2]-9.45667*snp7AA*f[2]-8.88593*snp7AR*f[2]-6.67764*snp8AR*f[2]
  -7.90793*snp9RR*f[2]-9.85191*snp10AA*f[2]-7.4805*snp1AA*f[3]-9.94246*snp1RR*f[3]
  +7.41821*snp3AA*f[3]+6.26351*snp3RR*f[3]-6.23167*snp4AA*f[3]+9.88171*snp5AA*f[3]
  +7.35601*snp5AR*f[3]+9.00426*snp6RR*f[3]-9.06503*snp7AA*f[3]-9.99652*snp7AR*f[3]
  -9.90821*snp7RR*f[3]-9.23499*snp8AR*f[3]+8.58147*snp8RR*f[3]-9.48169*snp9AA*f[3]
  -8.96275*snp10RR*f[3]+9.5994*snp1AR*f[4]-6.28187*snp1RR*f[4]+6.50309*snp2AA*f[4]
  -9.19834*snp2AR*f[4]-6.8201*snp3AR*f[4]+9.99739*snp3RR*f[4]+7.67176*snp4AA*f[4]
  -8.88641*snp4AR*f[4]-8.56208*snp4RR*f[4]-6.39933*snp5AR*f[4]+6.29145*snp6AA*f[4]
  -8.96704*snp6AR*f[4]+8.30926*snp7AR*f[4]-7.71597*snp8AA*f[4]+7.10362*snp8RR*f[4]
```

```
-9.88722*snp9AA*f[4]-7.68392*snp9AR*f[4]+6.88975*snp10AA*f[4]-9.808*snp10AR*f[4]
```

Grammatical evolution method

The method was developed recently to recover the analytic form of a function from known values [O'Neill and Ryan, 2001, Noorian et al., 2016].

Formal definition of a context-free grammar (CFG):

A *formal grammar* where every production rule, formalized by the pair (n, V), is in form of $n \to V$.

The CFG is defined by the 4-tuple $(\mathcal{T}, \mathcal{N}, \mathcal{R}, \mathcal{S})$, where

- $\mathcal{T}$ is the finite set of terminal symbols,
- $\mathcal{N}$ is the finite set of non-terminal symbols,
- $\mathcal{R}$ is the production rule set,
- $\mathcal{S} \in \mathcal{N}$ is the start symbol.

A production rule $n \to V$ is realized by replacing the non-terminal symbol $n \in \mathcal{N}$ with the symbol $v \in V$, where $V \in (\mathcal{T} \cup \mathcal{N})^*$ is a sequence of terminal and/or non-terminal symbols [Aho et al., 2006].

Using the following grammar:

Table S5: Example grammar.

<table>
<tr><td><expr></td><td>::=</td><td>(<expr>)<op>(<expr>) | <coef>*<var></td></tr>
<tr><td><op></td><td>::=</td><td>+ | - | * | /</td></tr>
<tr><td><coef></td><td>::=</td><td>c1 | c2</td></tr>
<tr><td><var></td><td>::=</td><td>v1 | v2</td></tr>
</table>

Example: **{0, 1, 0, 0, 1, 1, 1, 1}**

Table S6: Example of translation.

Step	Codon	Symbol	Rule	Result
0			starting:	<expr>
1	0	<expr>	(<expr>)<op>(<expr>)	(<expr>)<op>(<expr>)
2	1	<expr>	<coef>*<var>	(<coef>*<var>)<op>(<expr>)
3	0	<coef>	c1	(c1*<var>)<op>(<expr>)
4	0	<var>	v1	(c1*v1)<op>(<expr>)
5	1	<op>	-	(c1*v1)-(<expr>)
6	1	<expr>	<coef>*<var>	(c1*v1)-(<coef>*<var>)
7	1	<coef>	c2	(c1*v1)-(c2*<var>)
8	1	<var>	v2	**(c1*v1)-(c2*v2)**

The analytic representation of non-linear dependence of a agronomic traits on climatic factors is build using N functions from "words" of length L according to:

- allowed words: predictor's name or operation on expressions on predictors,
- allowed operations: '+', '-', '*', '/',
- allowed expressions: X, (X - Const), 1/(X - Const),

S4 Differential Evolution Entirely Parallel method

An effective stochastic method for function minimization termed Differential Evolution (DE), proposed in [Storn and Price, 1995], operates on a set (population) parameter vectors (individuals). The initial population is generated randomly, a size of population NP is fixed. DEEP one can be applied to solve both unconstrained and constrained optimization problems. Constraints may be imposed in the form of inequalities or equalities for a subset of parameters or their combinations.

DEEP method [Kozlov and Samsonov, 2011] incorporates the "trigonometric mutation" rule proposed in [Fan and Lampinen, 2003] and used to take into account a value of the objective function for each

individual at the recombination step, and the adaptive scheme for selection of internal parameters based on the control of the population diversity developed in [Zaharie, 2002].

In DEEP the age of individual is defined as a number of iterations, during which the individual survived without changes. The number of oldest individuals is substituted with the same number of the best ones after the predefined number of iterations to avoid local minima. Calculations are terminated when the objective function variation becomes less than a predefined value during the several consecutive steps or the maximal number of iterations is exceeded.

DE operates on floating point parameters, while two algorithms for parameter conversion from real to integer are implemented in DEEP [Kozlov et al., 2013]. The first method rounds off a real value to the nearest integer number. Another two step procedure firstly sorts parameters in ascending order and then uses the indices as integer parameters.

DEEP employs the pool of worker threads with asynchronous queue of tasks to evaluate the individual solutions in parallel. The calculation of objective function for each trial vector is pushed to the asynchronous queue and starts as soon as there is an available thread in the pool [Kozlov et al., 2016].

DEEP is implemented in C programming language as console application and using interfaces from GLIB project `https://developer.gnome.org/glib/`, e.g. *Thread Pool API*. DEEP method is an open source and free software distributed under the terms of GPL licence version 3. The sources are available at `https://gitlab.com/mackoel/deepmethod`.

References

[Aho et al., 2006] Aho, A. V., Lam, M. S., Sethi, R., and Ullman, J. D. (2006). *Compilers: Principles, Techniques, and Tools (2Nd Edition)*. Addison-Wesley Longman Publishing Co., Inc., Boston, MA, USA. 00000.

[Fan and Lampinen, 2003] Fan, H.-Y. and Lampinen, J. (2003). A Trigonometric Mutation Operation to Differential Evolution. *Journal of Global Optimization*, 27:25.

[Kozlov et al., 2013] Kozlov, K., Ivanisenko, N., Ivanisenko, V., Kolchanov, N., Samsonova, M., and Samsonov, A. M. (2013). Enhanced Differential Evolution Entirely Parallel Method for Biomedical Applications. In Hutchison, D., Kanade, T., Kittler, J., Kleinberg, J. M., Mattern, F., Mitchell, J. C., Naor, M., Nierstrasz, O., Pandu Rangan, C., Steffen, B., Sudan, M., Terzopoulos, D., Tygar, D., Vardi, M. Y., Weikum, G., and Malyshkin, V., editors, *Parallel Computing Technologies*, volume 7979, pages 409–416. Springer Berlin Heidelberg, Berlin, Heidelberg.

[Kozlov and Samsonov, 2011] Kozlov, K. and Samsonov, A. (2011). DEEP – Differential Evolution Entirely Parallel Method for Gene Regulatory Networks. *Journal of Supercomputing*, 57:172–178.

[Kozlov et al., 2016] Kozlov, K., Samsonov, A. M., and Samsonova, M. (2016). A software for parameter optimization with Differential Evolution Entirely Parallel method. *PeerJ Computer Science*, 2:e74.

[Noorian et al., 2016] Noorian, F., de Silva, A. M., and Leong, P. H. W. (2016). **gramEvol** : Grammatical Evolution in *R*. *Journal of Statistical Software*, 71(1):1–26.

[O'Neill and Ryan, 2001] O'Neill, M. and Ryan, C. (2001). Grammatical evolution. *IEEE Transactions on Evolutionary Computation*, 5(4):349–358.

[Storn and Price, 1995] Storn, R. and Price, K. (1995). Differential Evolution – A Simple and Efficient Heuristic for Global Optimization over Continuous Spaces. Technical Report Technical Report TR-95-012, ICSI.

[Zaharie, 2002] Zaharie, D. (2002). Parameter Adaptation in Differential Evolution by Controlling the Population Diversity. In Petcu, D., editor, *Proc. of 4th InternationalWorkshop on Symbolic and Numeric Algorithms for Scientific Computing*, volume XL of *Seria Matematica-Informatica*, pages 385–397, Timisoara, Romania. Analele Universitatii Timisoara.
